# Records of larentiine moths (Lepidoptera: Geometridae) collected at the Station Linné in Sweden

**DOI:** 10.3897/BDJ.4.e7304

**Published:** 2016-01-08

**Authors:** Olga Schmidt

**Affiliations:** ‡ SNSB-Zoologische Staatssammlung München, Munich, Germany

**Keywords:** Alvar, checklist, diversity, faunistics, geometrid moths, Larentiinae, malaise trap sampling, Öland, Skogsby

## Abstract

**Background:**

The island of Öland, at the southeast of Sweden, has unique geological and environmental features. The Station Linné is a well-known Öland research station which provides facilities for effective studies and attracts researchers from all over the world. Moreover, the station remains a center for ecotourism due to extraordinary biodiversity of the area. The present paper is aimed to support popular science activities carried out on the island and to shed light on diverse geometrid moth fauna of the Station Linné.

**New information:**

As an outcome of several research projects, including the Swedish Malaise Trap Project (SMTP) and the Swedish Taxonomy Initiative (STI) conducted at the Station Linné, a list of larentiine moths (Lepidoptera: Geometridae) collected on the territory of the station is presented. Images of moths from above and underside are shown. Of the totally 192 species registered for Sweden, 41 species (more than 21%) were collected in close proximity to the main building of the Station Linné. Malaise trap sampling of Lepidoptera is discussed.

## Introduction

The island of Öland, at the southeast of Sweden, is famous for its dominant environmental feature, an Ordovician limestone pavement, which is called the Stora Alvaret (= the Great Alvar). Alvars are semi-natural grasslands which have been formed and developed due to long periods of human influence, including grazing (Rosén 1982). The Stora Alvaret has been designated as a World Heritage Site by UNESCO due to its unusual biodiversity and prehistory. Station Linné is situated on the island of Öland being a center for research, popular science activities, and ecotourism. The research station is named after Carl von Linné who visited Öland in 1741.

The Swedish Malaise Trap Project (SMTP), funded by the Swedish Species Information Centre (ArtDatabanken), is based at the Station Linné. The project aims to provide species determinations for the specimens obtained from Malaise traps sampling at a wide range of landscapes and habitats. For many groups, including geometrid moths, the final data release is still awaited. The present paper is aimed to present a first list of the larentiine moths collected at the Station Linné.

## Materials and methods

**Figure 1.**
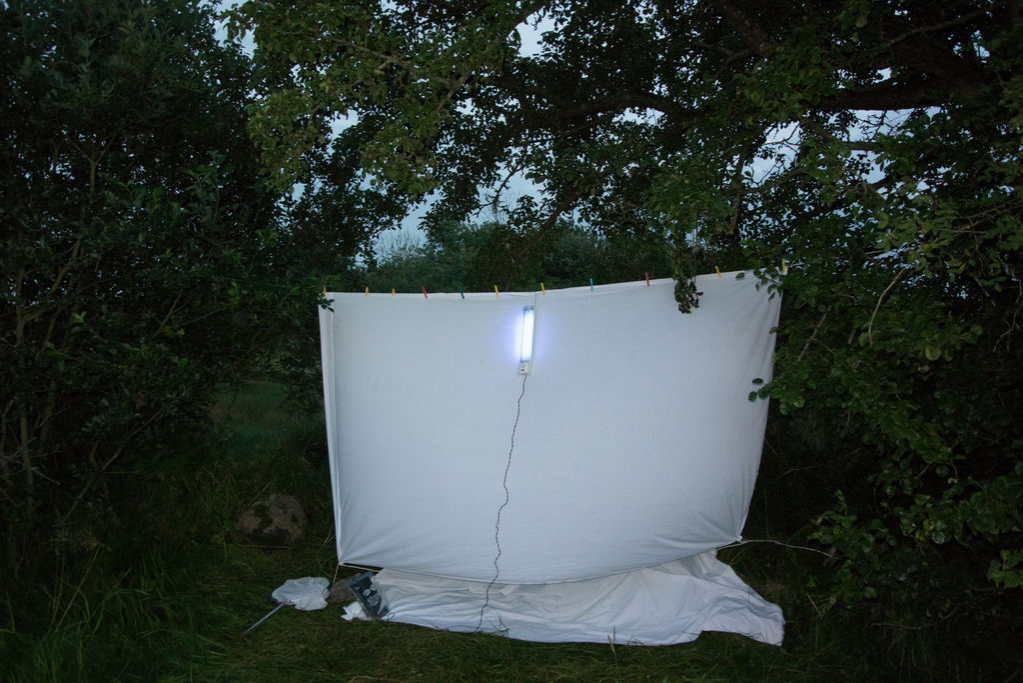
UV light trap

**Figure 2.**
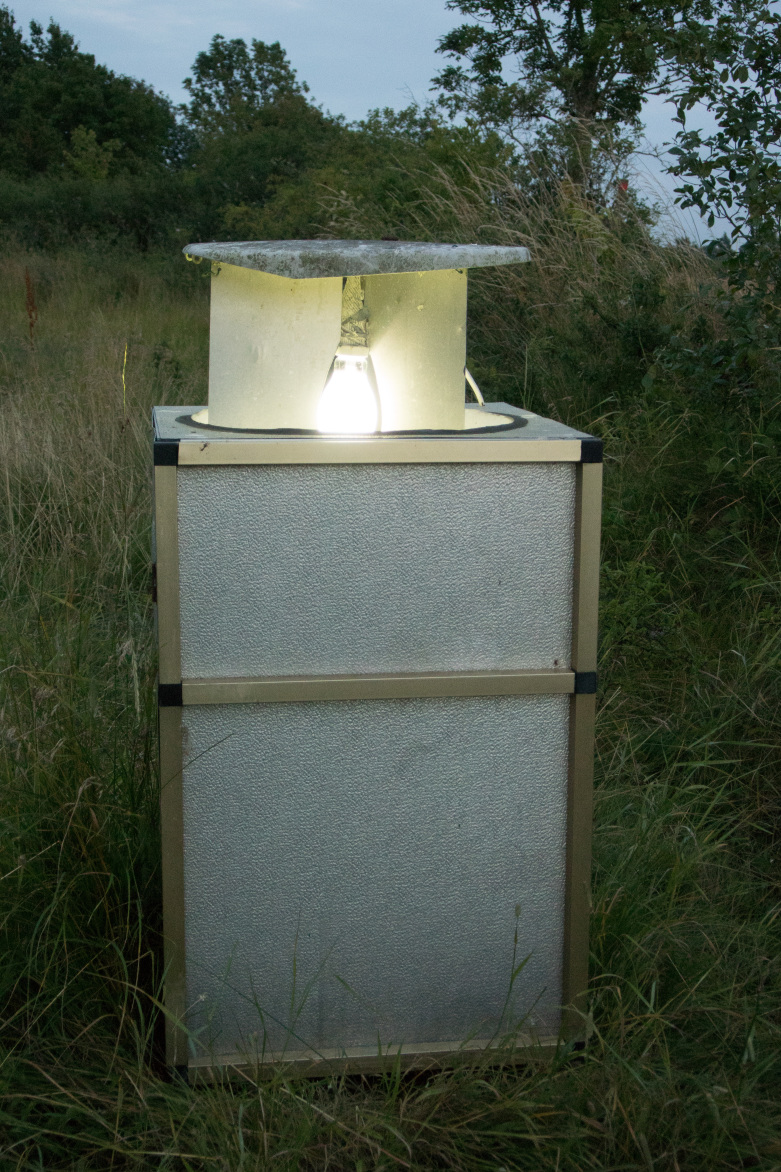
MV light trap

Material for study was mainly collected using a UV light trap (UV), a mercury vapor light trap (MV) and net sweeping (NS) by O. Schmidt in 2014 (June 24-29, July 1-4) and 2015 (July 20-31) in the Mörbylånga kommun, Skogsby, Station Linné (56.6186 N, 16.4989 E). The UV light trap was placed between the tree and shrub rows along a walking path, with a meadow on one side and a swampy area on the other side (Fig. 1). The mercury vapor light trap was situated nearby, at the edge of the swamp (Fig. 2). The two light traps were separated by a row of trees and shrubs.

Furthermore, material collected as part of the SMTP in 2007 and 2008 using a Malaise trap (MF) located close to the main building of the Station Linné (see http://www.stationlinne.se/sv/forskning/the-swedish-malaise-trap-project-smtp/traps/trap-id-2006-skogs, Trap ID 2006) was checked and the larentiine moths identified. This Malaise trap was placed on a lawn, about 100 m north of the Alvar edge (56.6190 N, 16.4973 E) and was running from April 2007 until November 2008. A note is given for the species recorded from Malaise trap samples only.

Additional material has been collected in the following locations: Borgholms kommun, Ismantorp; Borgholms kommun, Petgärdeträsk; Mörbylånga kommun, near Arontorp; Mörbylånga kommun, Gårdby; Mörbylånga kommun, Gillsättra_wet, Gillsättra_dry; Mörbylånga kommun, Jordtorpsåsen_wet, Jordtorpsåsen_dry; Mörbylånga kommun, Kalkstad; Mörbylånga kommun, Södra Sandby (Suppl. material 1). Specimens collected by net sweeping on Öland outside the station are marked with asterisk (NS*).

The genitalia of all small-sized moths were studied to correctly identify the species. The material was identified using the Lepidoptera collection of the Zoologische Staatssammlung München (ZSM, Germany) and publications by Mironov (2003), Elmquist et al. (2011) and Hausmann and Viidalepp (2012).

## Checklists

### List of larentiine moth species collected at the Station Linné

#### Phibalapteryx virgata

(Hufnagel, 1767)

http://eol.org/pages/4031647/overview

http://www.lepiforum.de/lepiwiki.pl?Phibalapteryx_virgata

##### Notes

Figs 3, 4

#### Cidaria fulvata

(Forster, 1771)

http://eol.org/pages/283762/overview

http://www.lepiforum.de/lepiwiki.pl?Cidaria_Fulvata

##### Notes

Figs 5, 6

#### Colostygia olivata

(Denis & Schiffermüller, 1775)

http://eol.org/pages/277279/overview

http://www.lepiforum.de/lepiwiki.pl?Colostygia_olivata

##### Notes

Figs 7, 8

#### Colostygia pectinataria

(Knoch, 1781)

http://eol.org/pages/278481/overview

http://www.lepiforum.de/lepiwiki.pl?Colostygia_Pectinataria

##### Notes

Figs 9, 10

#### Cosmorhoe ocellata

(Linnaeus, 1758)

http://eol.org/pages/270324/overview

http://www.lepiforum.de/lepiwiki.pl?Cosmorhoe_Ocellata

##### Notes

Figs 11, 12

#### Eulithis prunata

(Linnaeus, 1758)

http://eol.org/pages/281849/overview

http://www.lepiforum.de/lepiwiki.pl?Eulithis_Prunata

##### Notes

Figs 13, 14

#### Eulithis mellinata

(Fabricius, 1787)

http://eol.org/pages/284564/overview

http://www.lepiforum.de/lepiwiki.pl?Eulithis_Mellinata

##### Notes

Figs 15, 16

#### Eulithis testata

(Linnaeus, 1761)

http://eol.org/pages/286201/overview

http://www.lepiforum.de/lepiwiki.pl?Eulithis_Testata

##### Notes

Figs 17, 18

#### Gandaritis pyraliata

(Denis & Schiffermüller, 1775)

http://eol.org/pages/4017307/overview

http://www.lepiforum.de/lepiwiki.pl?Gandaritis_Pyraliata

##### Notes

Figs 19, 20

#### Plemyria rubiginata

(Denis & Schiffermüller, 1775)

http://eol.org/search?q=Plemyria+rubiginata&search=Go

http://www.lepiforum.de/lepiwiki.pl?Plemyria_Rubiginata

##### Notes

Figs 21, 22

#### Thera cognata

(Thunberg, 1792)

http://eol.org/pages/298019/overview

http://www.lepiforum.de/lepiwiki.pl?Thera_cognata

##### Notes

Figs 23, 24

#### Eupithecia absinthiata

(Clerck, 1759)

http://eol.org/pages/285123/overview

http://www.lepiforum.de/lepiwiki.pl?Eupithecia_absinthiata

http://mothphotographersgroup.msstate.edu/species.php?hodges=7586.1

##### Notes

Figs 25, 26

#### Eupithecia denotata

(Hübner, 1813)

http://eol.org/search?q=Eupithecia+denotata&search=Go

http://www.lepiforum.de/lepiwiki.pl?Eupithecia_denotata

http://www.euroleps.ch/seiten/s_art.php?art=geo_denotata

##### Notes

Figs 27, 28

#### Eupithecia exiguata

(Hübner, 1813)

http://eol.org/pages/284131/overview

http://www.lepiforum.de/lepiwiki.pl?Eupithecia_exiguata

##### Notes

Figs 29, 30

#### Eupithecia icterata

(de Villers, 1789)

http://eol.org/pages/281026/overview

http://www.lepiforum.de/lepiwiki.pl?Eupithecia_icterata

##### Notes

Figs 31, 32

#### Eupithecia linariata

(Denis & Schiffermüller, 1775)

http://eol.org/pages/283937/overview

http://www.lepiforum.de/lepiwiki.pl?Eupithecia_Linariata

##### Notes

Figs 33, 34

#### Eupithecia nanata

(Hübner, 1813)

http://eol.org/pages/287885/overview

http://www.lepiforum.de/lepiwiki.pl?Eupithecia_nanata

##### Notes

Figs 35, 36

#### Eupithecia pusillata

(Denis & Schiffermüller, 1775)

http://eol.org/search?http://eol.org/search?q=Eupithecia+pusillata&search=Go

http://www.lepiforum.de/lepiwiki.pl?Eupithecia_Pusillata

##### Notes

Figs 37, 38

#### Eupithecia satyrata

(Hübner, 1813)

http://eol.org/pages/292707/overview

http://www.lepiforum.de/lepiwiki.pl?Eupithecia_satyrata

##### Notes

Figs 39, 40

#### Eupithecia subfuscata

(Haworth, 1809)

http://eol.org/pages/286559/overview

http://www.lepiforum.de/lepiwiki.pl?Eupithecia_Subfuscata

##### Notes

Figs 41, 42

#### Eupithecia subumbrata

(Denis & Schiffermüller, 1775)

http://eol.org/pages/287281/overview

http://www.lepiforum.de/lepiwiki.pl?Eupithecia_subumbrata

##### Notes

Figs 43, 44

#### Eupithecia succenturiata

(Linnaeus, 1758)

http://eol.org/pages/292925/overview

http://www.lepiforum.de/lepiwiki.pl?Eupithecia_succenturiata

##### Notes

Figs 45, 46

#### Eupithecia tenuiata

(Hübner, 1813)

http://eol.org/pages/285093/overview

http://www.lepiforum.de/lepiwiki.pl?Eupithecia_tenuiata

##### Notes

Figs 47, 48

#### Eupithecia valerianata

(Hübner, 1813)

https://en.wikipedia.org/wiki/Eupithecia_valerianata

http://www.lepiforum.de/lepiwiki.pl?Eupithecia_Valerianata

##### Notes

Figs 49, 50

#### Pasiphila chloerata

(Mabille, 1870)

http://eol.org/pages/4031644/overview

http://www.lepiforum.de/lepiwiki.pl?Pasiphila_Chloerata

##### Notes

Figs 51, 52

#### Pasiphila rectangulata

(Linnaeus, 1758)

http://eol.org/pages/277386/overview

http://www.lepiforum.de/lepiwiki.pl?Pasiphila_Rectangulata

##### Notes

Figs 53, 54

#### Hydriomena furcata

(Thunberg, 1784)

http://eol.org/pages/286763/overview

http://www.lepiforum.de/lepiwiki.pl?Hydriomena_furcata

##### Notes

Figs 55, 56

#### Pelurga comitata

(Linnaeus, 1758)

http://eol.org/pages/284799/overview

http://www.lepiforum.de/lepiwiki.pl?Pelurga_Comitata

##### Notes

Figs 57, 58

#### Mesotype didymata

(Linnaeus, 1758)

http://eol.org/pages/4012784/overview

http://www.lepiforum.de/lepiwiki.pl?Mesotype_Didymata

##### Notes

Figs 59, 60

#### Perizoma alchemillata

(Linnaeus, 1758)

http://eol.org/pages/295004/overview

http://www.lepiforum.de/lepiwiki.pl?Perizoma_Alchemillata

##### Notes

Figs 61, 62

#### Philereme transversata

(Hufnagel, 1767)

http://eol.org/pages/296282/overview

http://www.lepiforum.de/lepiwiki.pl?Philereme_transversata

##### Notes

Figs 63, 64

#### Philereme vetulata

(Denis & Schiffermüller, 1775)

http://eol.org/search?http://eol.org/search?q=Philereme+vetulata&search=Go

http://www.lepiforum.de/lepiwiki.pl?Philereme_vetulata

##### Notes

Figs 65, 66

#### Scotopteryx chenopodiata

(Linnaeus, 1758)

http://eol.org/pages/295986/overview

http://www.lepiforum.de/lepiwiki.pl?Scotopteryx_Chenopodiata

##### Notes

Figs 67, 68

#### Pterapherapteryx sexalata

(Retzius, 1783)

http://eol.org/pages/297593/overview

http://www.lepiforum.de/lepiwiki.pl?Pterapherapteryx_sexalata

##### Notes

Figs 69, 70

#### Camptogramma bilineata

(Linnaeus, 1758)

http://eol.org/search?http://eol.org/search?q=Camptogramma+bilineata&search=Go

http://www.lepiforum.de/lepiwiki.pl?Camptogramma_Bilineata

##### Notes

Figs 71, 72

#### Catarhoe cuculata

(Hufnagel, 1767)

http://eol.org/pages/276073/overview

http://www.lepiforum.de/lepiwiki.pl?Catarhoe_cuculata

##### Notes

Figs 73, 74

#### Catarhoe rubidata

(Denis & Schiffermüller, 1775)

http://eol.org/pages/279041/overview

http://www.lepiforum.de/lepiwiki.pl?Catarhoe_Rubidata

##### Notes

Figs 75, 76

#### Epirrhoe alternata

(Müller, 1764)

http://eol.org/search?q=Epirrhoe+alternata&search=Go

http://www.lepiforum.de/lepiwiki.pl?Epirrhoe_Alternata

##### Notes

Figs 77, 78

#### Epirrhoe hastulata

(Hübner, 1790)

http://eol.org/pages/285918/overview

http://www.lepiforum.de/lepiwiki.pl?Epirrhoe_Hastulata

##### Notes

Figs 79, 80

#### Epirrhoe tristata

(Linnaeus, 1758)

http://eol.org/pages/285474/overview

http://www.lepiforum.de/lepiwiki.pl?Epirrhoe_Tristata

##### Notes

Figs 81, 82

#### Xanthorhoe ferrugata

(Clerck, 1759)

http://eol.org/pages/288630/overview

http://www.lepiforum.de/lepiwiki.pl?Xanthorhoe_ferrugata

##### Notes

Figs 83, 84

## Discussion

### Results and discussion

Totally, 192 species of Larentiinae are recorded for Sweden (Elmquist et al. 2011, http://www2.nrm.se/en/svenska_fjarilar/svenska_fjarilar.html). Bert Gustafsson listed 156 species occurring on Öland (http://www2.nrm.se/en/catalogus.html.se). Currently, 41 species are recorded for the territory of the Station Linné, which comprises 26.3% of the Öland species and more than 21% of the Swedish larentiine fauna. Interestingly, 37 species were sampled during 22 nights of light trapping in summer 2014 and 2015, when the weather was not quite favorable for collecting. For comparison, a recent rapid biotic survey at a 365 hectare Charitable Research Reserve in Ontario (Canada) revealed only nine larentiine species (Telfer et al. 2015). An unusual biodiversity registered for a small collecting site on Öland can be explained by use of effective sampling methods.

Most of the larentiine species were collected using a UV light trap. The exceptions are as follows: one specimen of *Eulithis testata* and one specimen of *Catarhoe cuculata* were attracted only to the Mercury vapor lamp. The efficiency of different types of traps in this study should be compared with caution. The Mercury vapor trap and the surrounding vegetation was checked once at night between 11 p.m. and 12 p.m. and emptied in the morning, after completion of light trapping, whereas the UV trap has been checked continuously and the geometrid moths flying near the trap and sitting on the leaves of trees and bushes were collected permanently. *Epirrhoe hastulata*, *E. tristata*, *Eupithecia exiguata* and *E. satyrata* were recorded only in Malaise trap samples collected during July 24 – August 12, 2008, May 12 – June 5, 2008, June 5-21, 2008, and June 1-15, 2007 respectively.

### Morphological differences within *Gandaritis pyraliata* (Denis & Schiffermüller)

A series of specimens presumably belonging to the species *Gandaritis pyraliata* have been collected. The specimens display variation in the wing pattern above and underneath, in the male genitalia (the shape of the saccus) and in the female genitalia (the length of the ductus bursae and the shape of the signum). The specimens require more detailed study.

### Malaise trap sampling of Lepidoptera

Malaise traps are effectively used for collecting small flying insects for many decades, after the trap has been described by Malaise (1937). Although butterflies and moths are sometimes target groups of large-scale malaise trap sampling for ecological and conservation studies (*e.g.* Basset et al. 2007, Campbell and Hanula 2007, Lamarre et al. 2012), collecting Lepidoptera by means of malaise traps is a challenging method. Designed for Diptera and Hymenoptera, a malaise trap indeed effectively samples Lepidoptera, as they get trapped within the malaise tent, flying upward towards either the sun (during the day) or the moon (at night) (see Lamarre et al. 2012). However, the specimens fall into a collecting jar filled with Ethanol, whereby the wing scales rub off easily. Generally, only specimens with distinct wing pattern can be reliably identified from the samples in Ethanol. The older the samples are, the more difficult it is to get a correct identification of Lepidoptera. For small moths it is necessary to study the genitalia or to perform a molecular analysis. Considering the results of present study, using only malaise traps for sampling Lepidoptera is advisable for well-studied faunas. Traditional methods, like net sweeping, light trapping or bait-traps deliver more suitable results.

## Supplementary Material

Supplementary material 1 List of larentiine moth species (Lepidoptera: Geometridae) of the Station Linné Data type: occurences
File: oo_64905.xls Schmidt, O.

XML Treatment for Phibalapteryx virgata

XML Treatment for Cidaria fulvata

XML Treatment for Colostygia olivata

XML Treatment for Colostygia pectinataria

XML Treatment for Cosmorhoe ocellata

XML Treatment for Eulithis prunata

XML Treatment for Eulithis mellinata

XML Treatment for Eulithis testata

XML Treatment for Gandaritis pyraliata

XML Treatment for Plemyria rubiginata

XML Treatment for Thera cognata

XML Treatment for Eupithecia absinthiata

XML Treatment for Eupithecia denotata

XML Treatment for Eupithecia exiguata

XML Treatment for Eupithecia icterata

XML Treatment for Eupithecia linariata

XML Treatment for Eupithecia nanata

XML Treatment for Eupithecia pusillata

XML Treatment for Eupithecia satyrata

XML Treatment for Eupithecia subfuscata

XML Treatment for Eupithecia subumbrata

XML Treatment for Eupithecia succenturiata

XML Treatment for Eupithecia tenuiata

XML Treatment for Eupithecia valerianata

XML Treatment for Pasiphila chloerata

XML Treatment for Pasiphila rectangulata

XML Treatment for Hydriomena furcata

XML Treatment for Pelurga comitata

XML Treatment for Mesotype didymata

XML Treatment for Perizoma alchemillata

XML Treatment for Philereme transversata

XML Treatment for Philereme vetulata

XML Treatment for Scotopteryx chenopodiata

XML Treatment for Pterapherapteryx sexalata

XML Treatment for Camptogramma bilineata

XML Treatment for Catarhoe cuculata

XML Treatment for Catarhoe rubidata

XML Treatment for Epirrhoe alternata

XML Treatment for Epirrhoe hastulata

XML Treatment for Epirrhoe tristata

XML Treatment for Xanthorhoe ferrugata

## Figures and Tables

**Figure 3.**
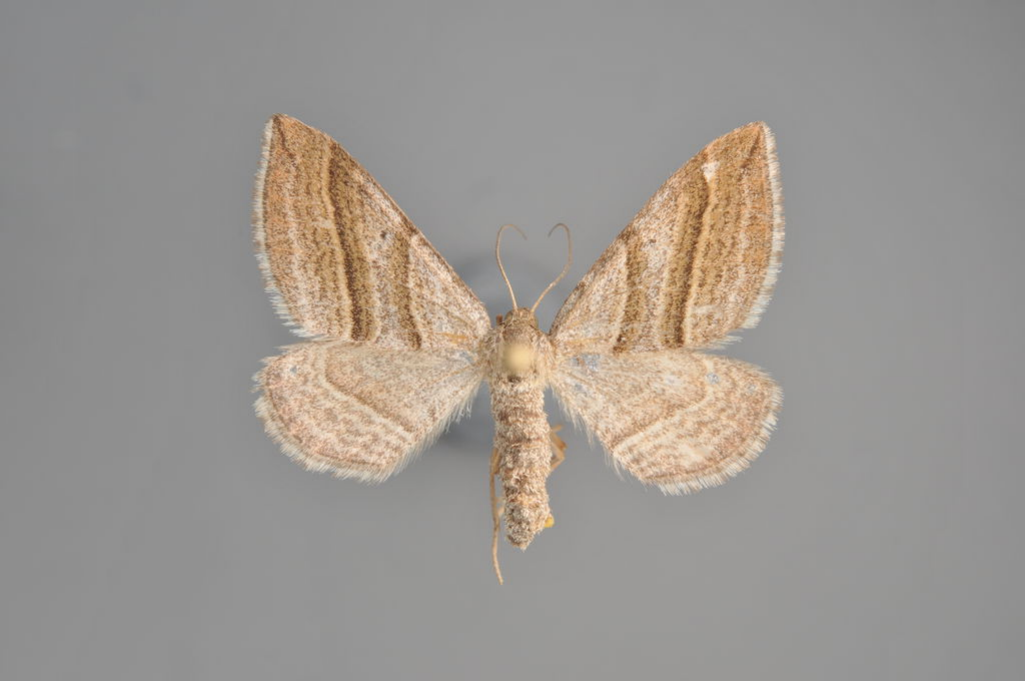
*Phibalapteryx virgata*, above

**Figure 4.**
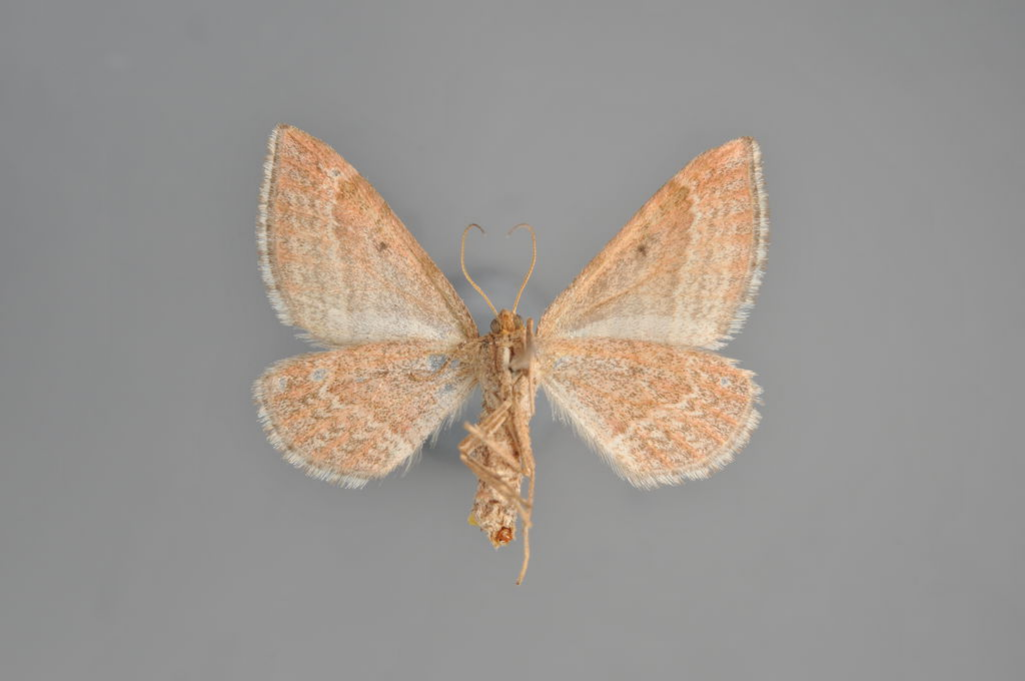
*Phibalapteryx virgata*, underneath

**Figure 5.**
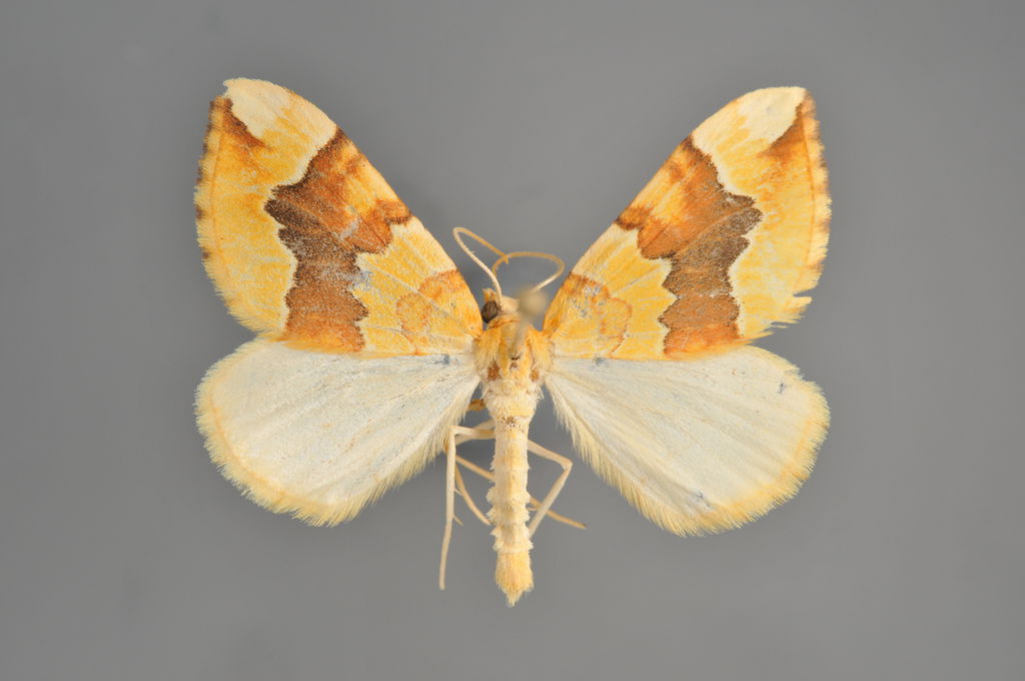
*Cidaria fulvata*, above

**Figure 6.**
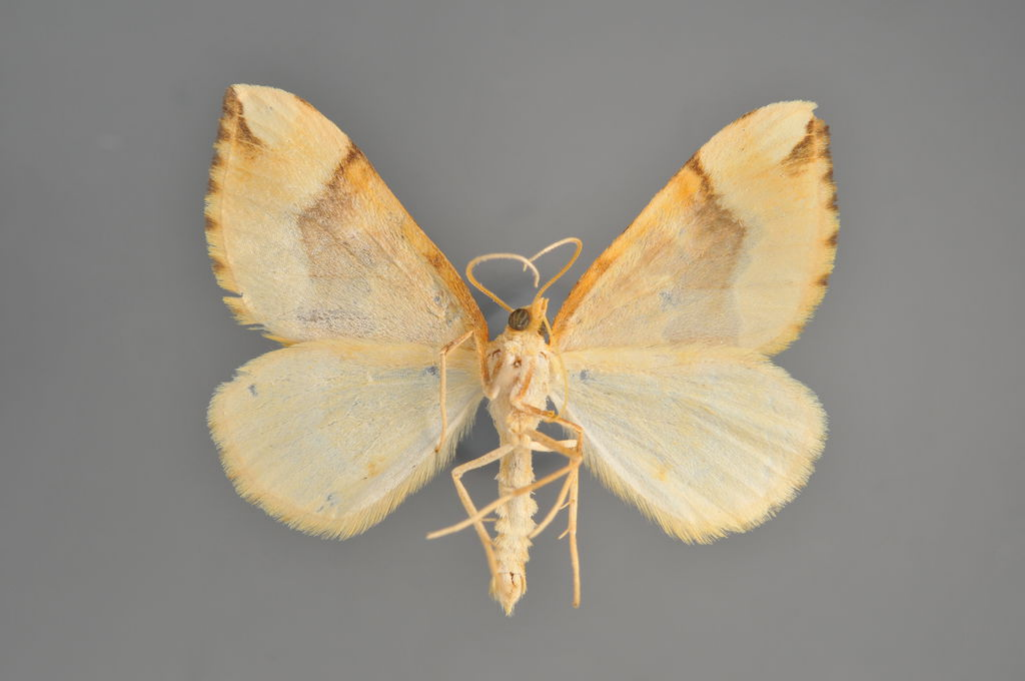
*Cidaria fulvata*, underneath

**Figure 7.**
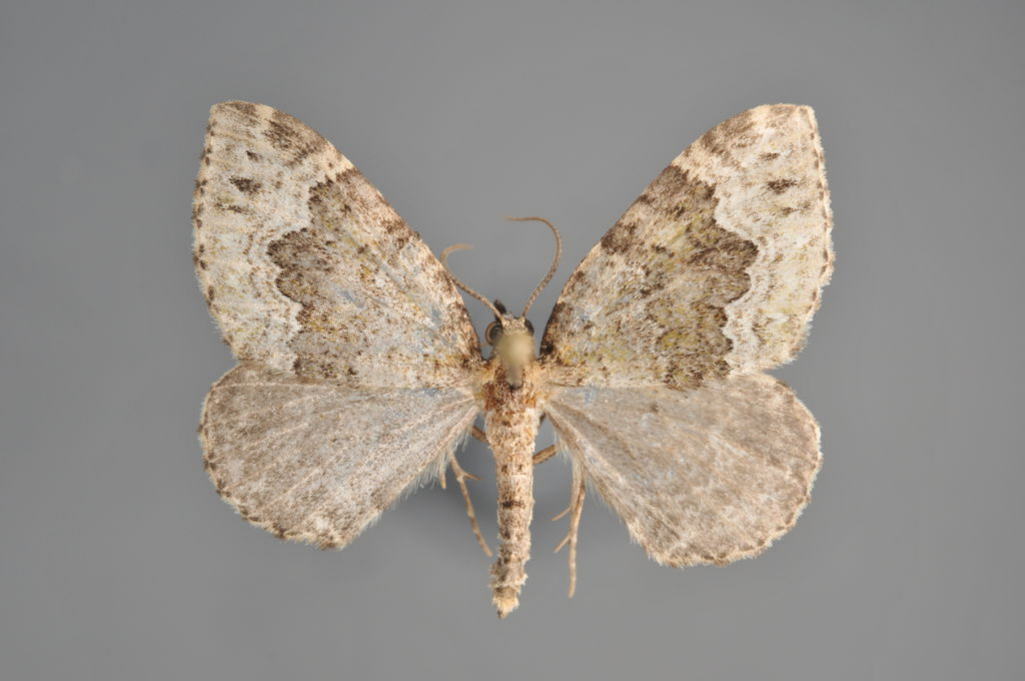
*Colostygia olivata*, above

**Figure 8.**
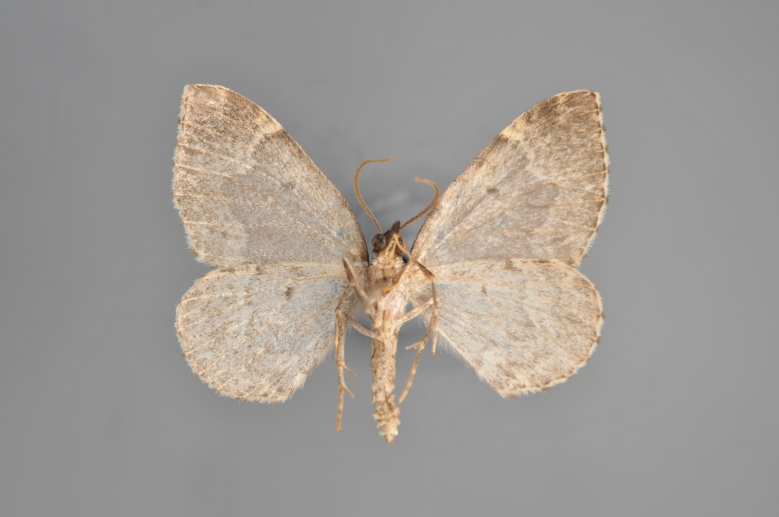
*Colostygia olivata*, underneath

**Figure 9.**
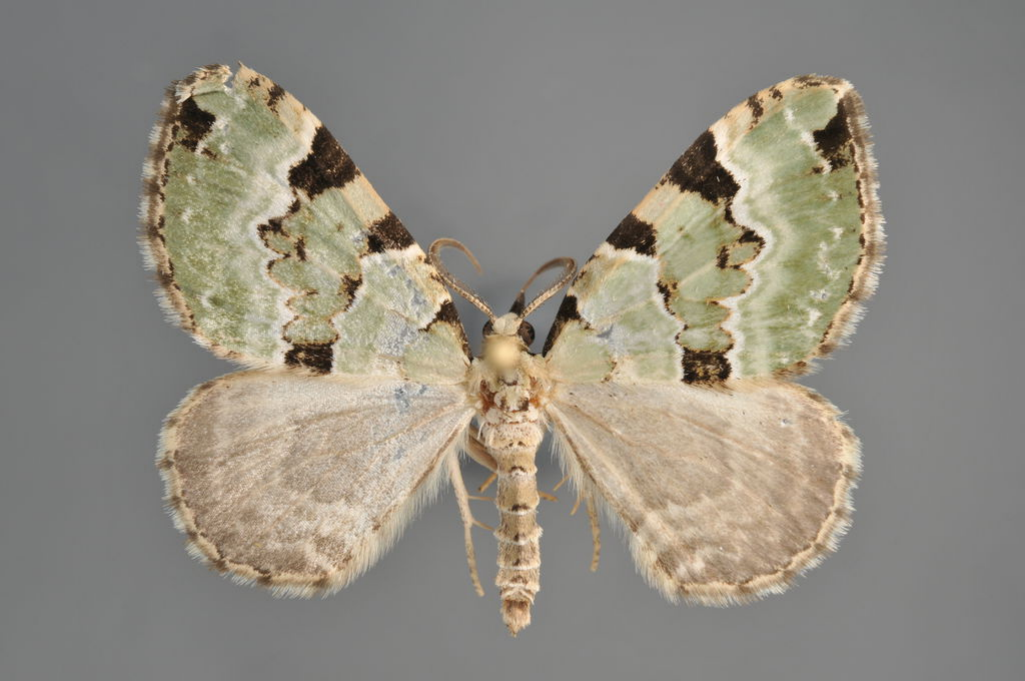
*Colostygia pectinataria*, above

**Figure 10.**
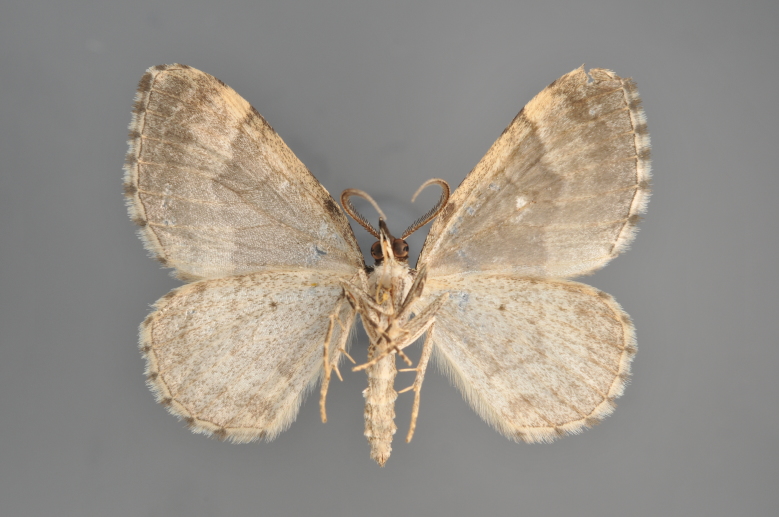
*Colostygia pectinataria*, underneath

**Figure 11.**
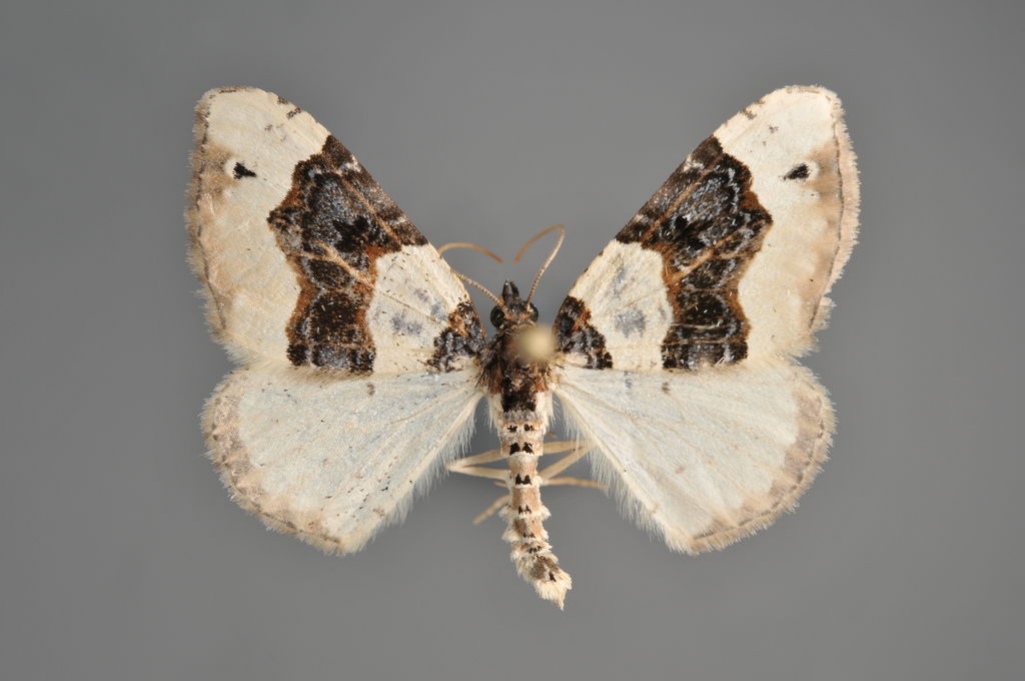
*Cosmorhoe ocellata*, above

**Figure 12.**
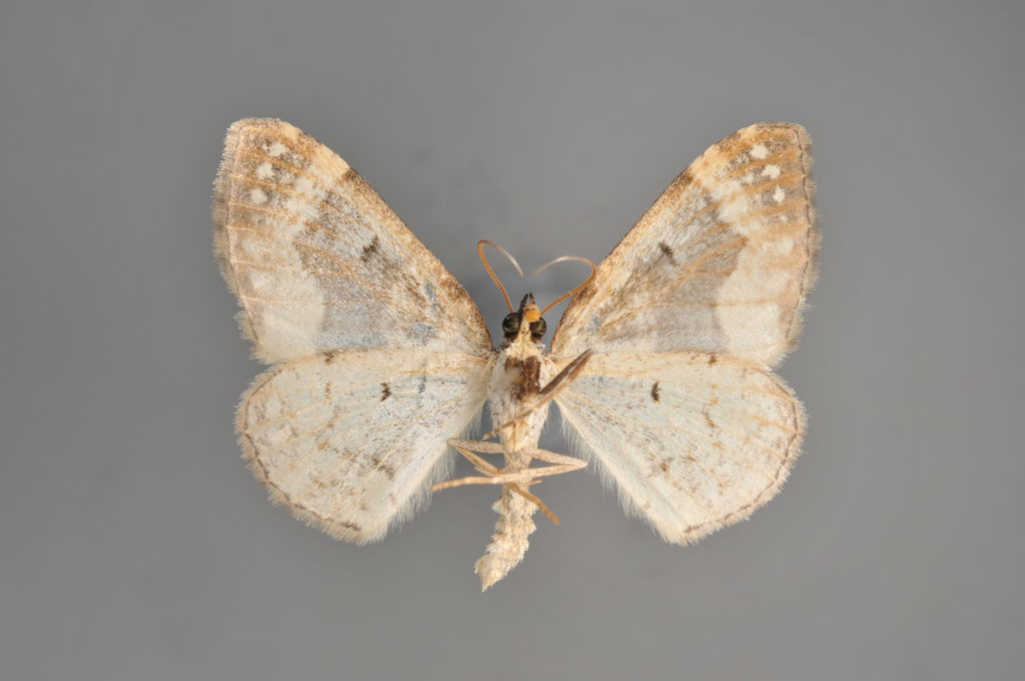
*Cosmorhoe ocellata*, underneath

**Figure 13.**
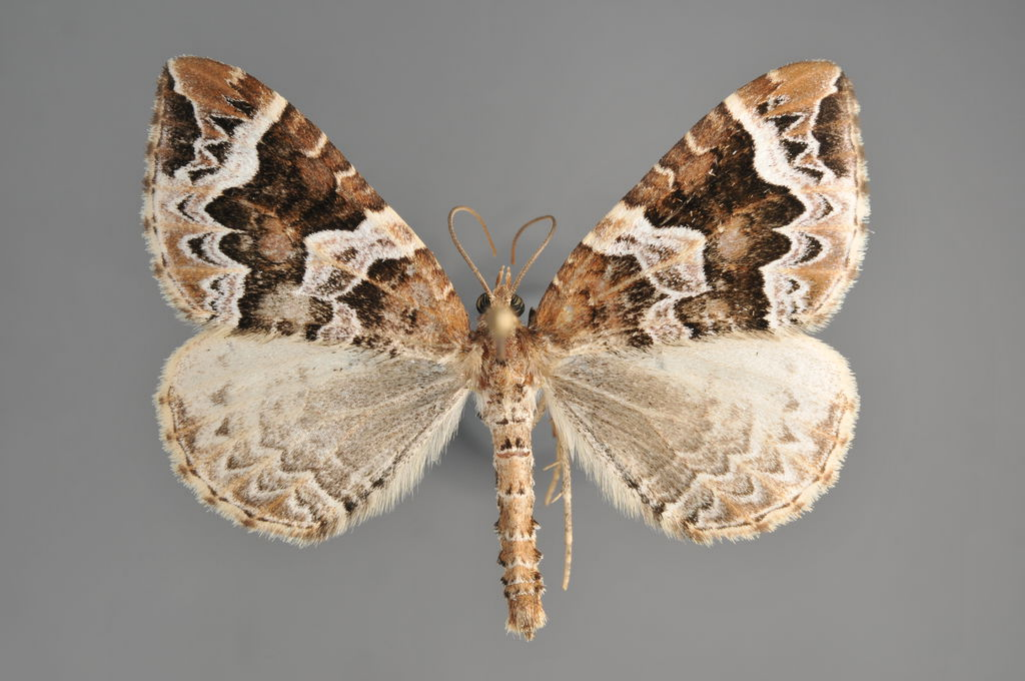
*Eulithis prunata*, above

**Figure 14.**
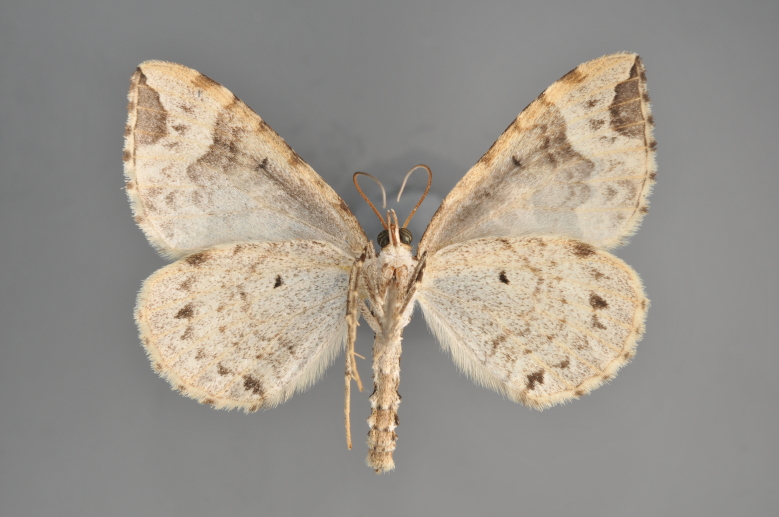
*Eulithis prunata*, underneath

**Figure 15.**
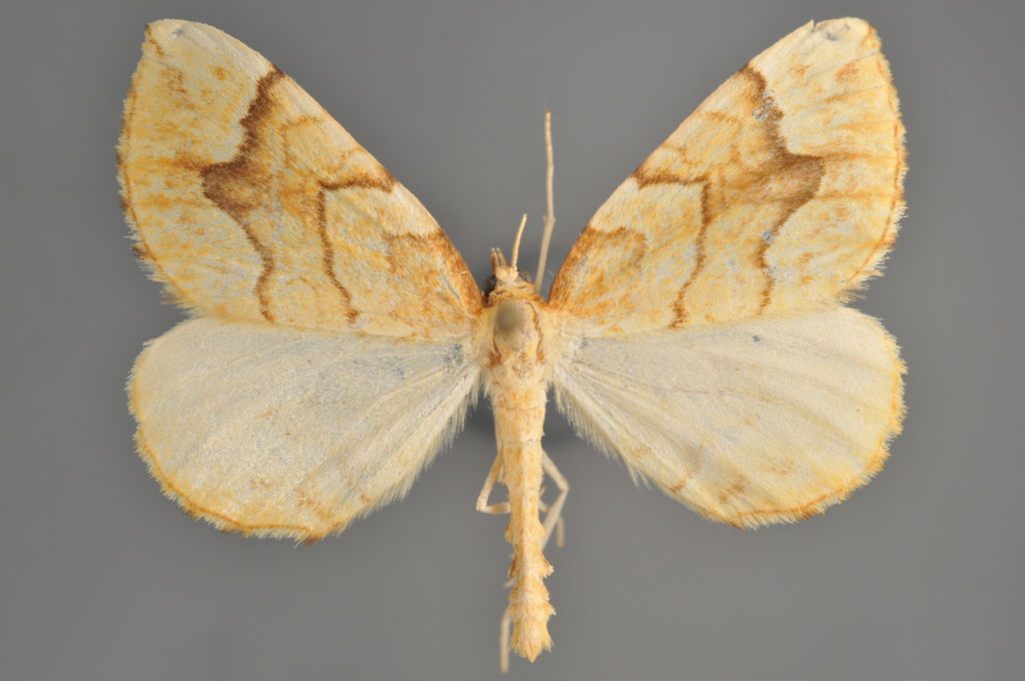
*Eulithis mellinata*, above

**Figure 16.**
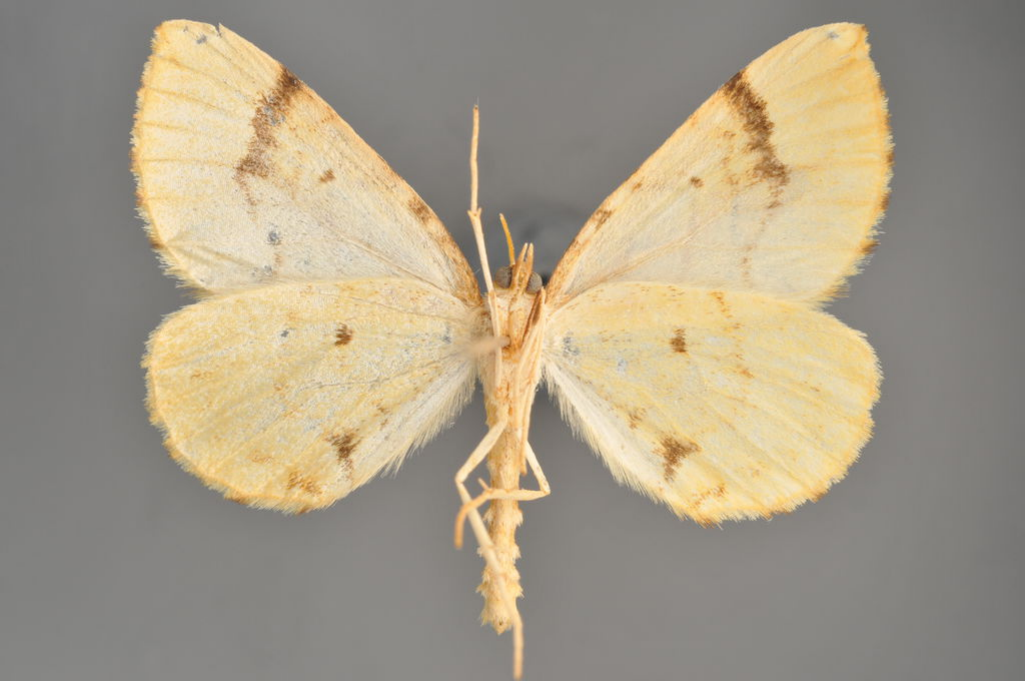
*Eulithis mellinata*, underneath

**Figure 17.**
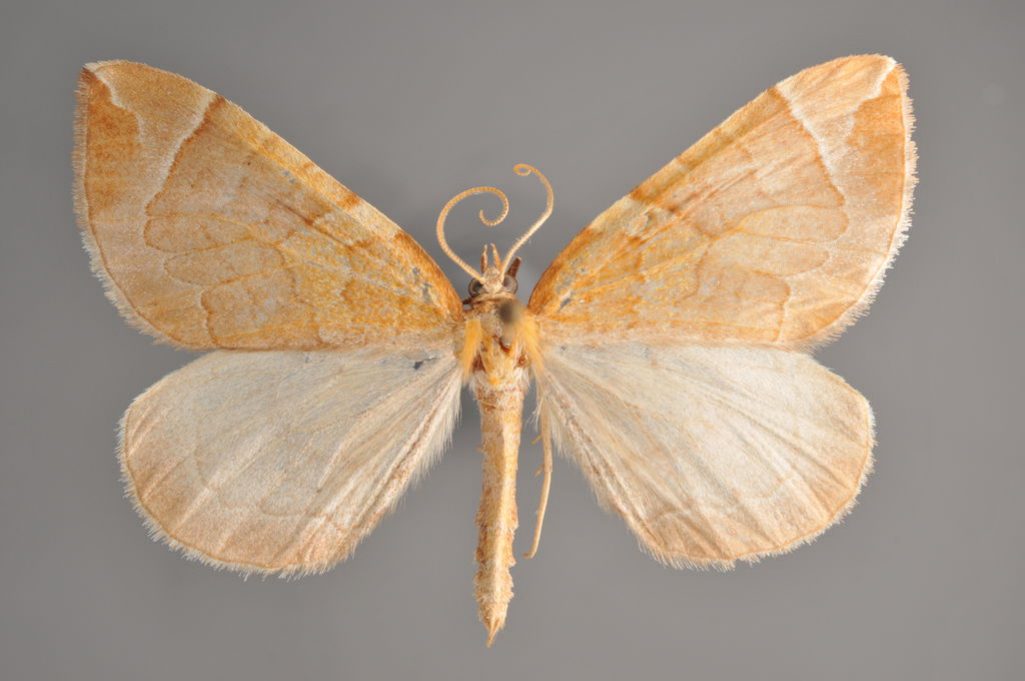
*Eulithis testata*, above

**Figure 18.**
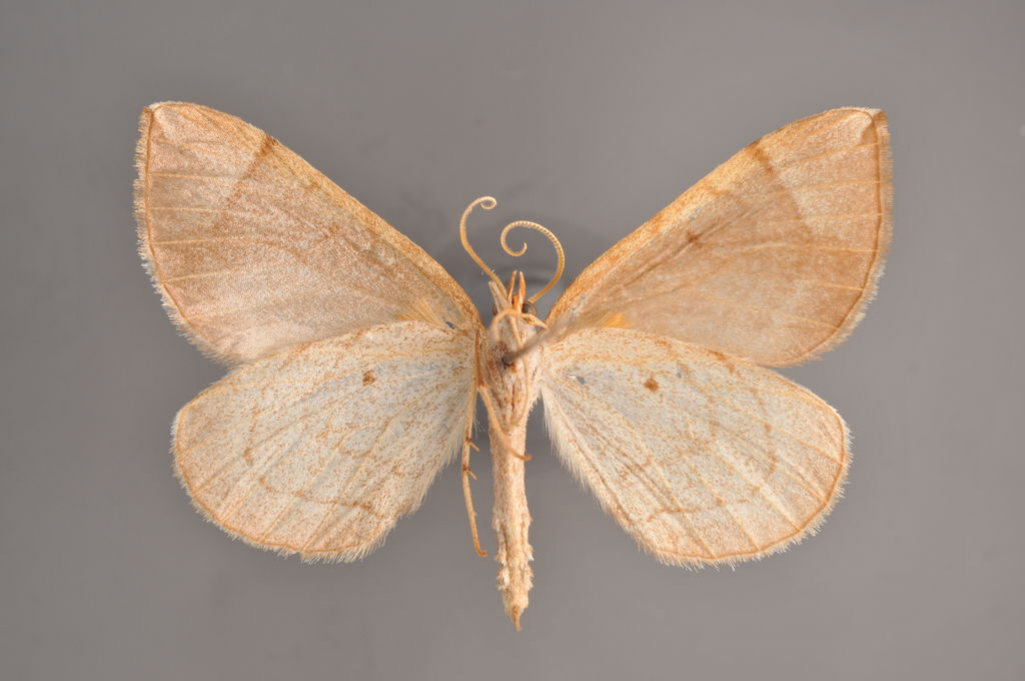
*Eulithis testata*, underneath

**Figure 19.**
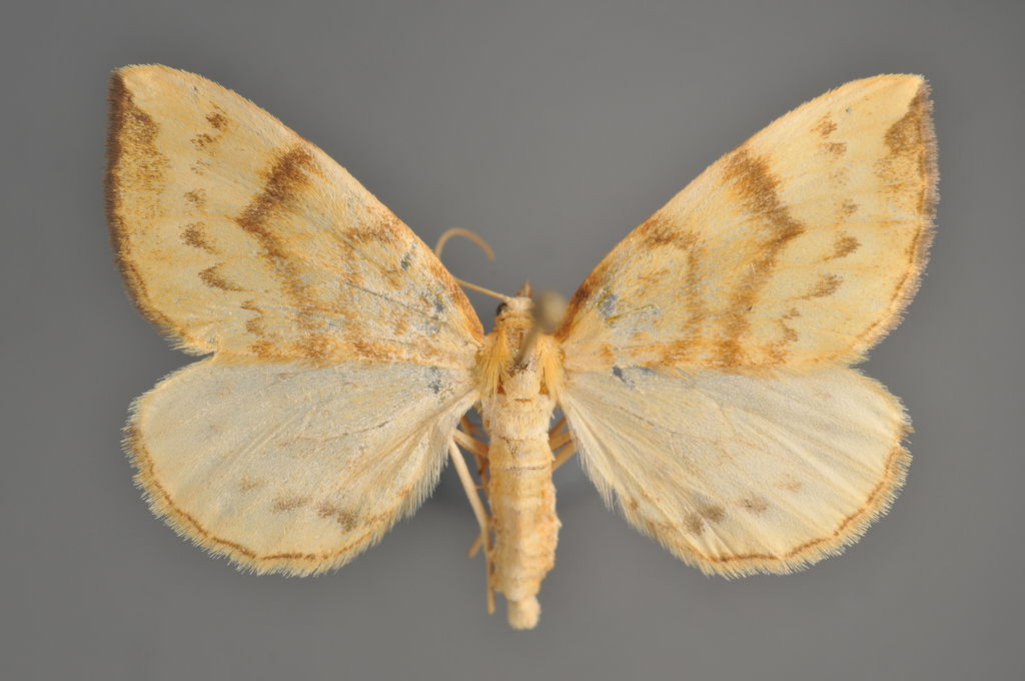
*Gandaritis pyraliata*, above

**Figure 20.**
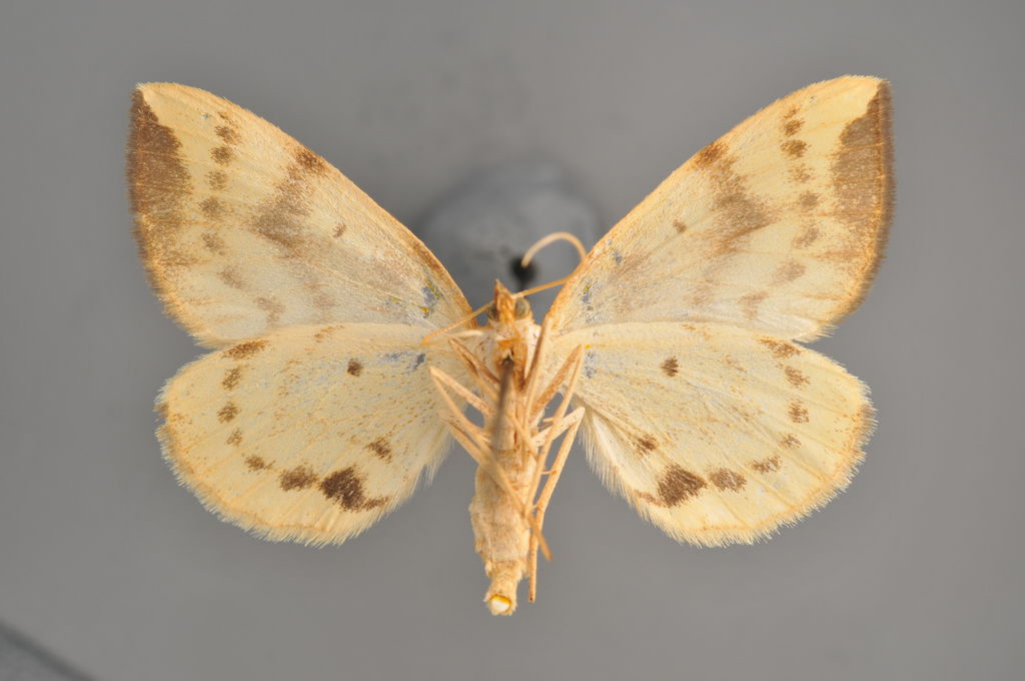
*Gandaritis pyraliata*, underneath

**Figure 21.**
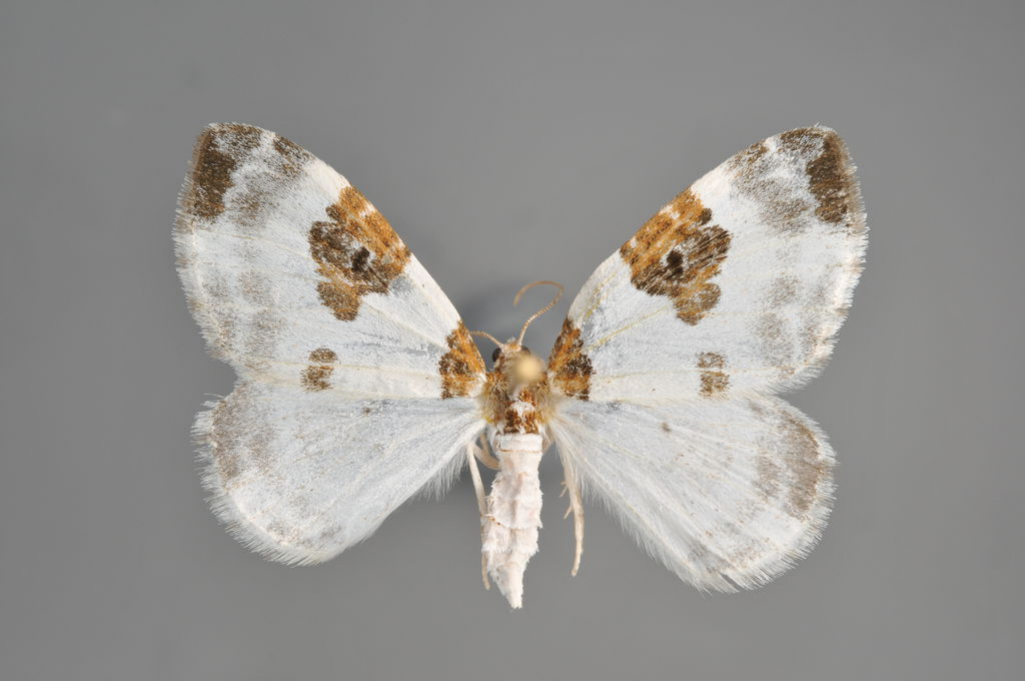
*Plemyria rubiginata*, above

**Figure 22.**
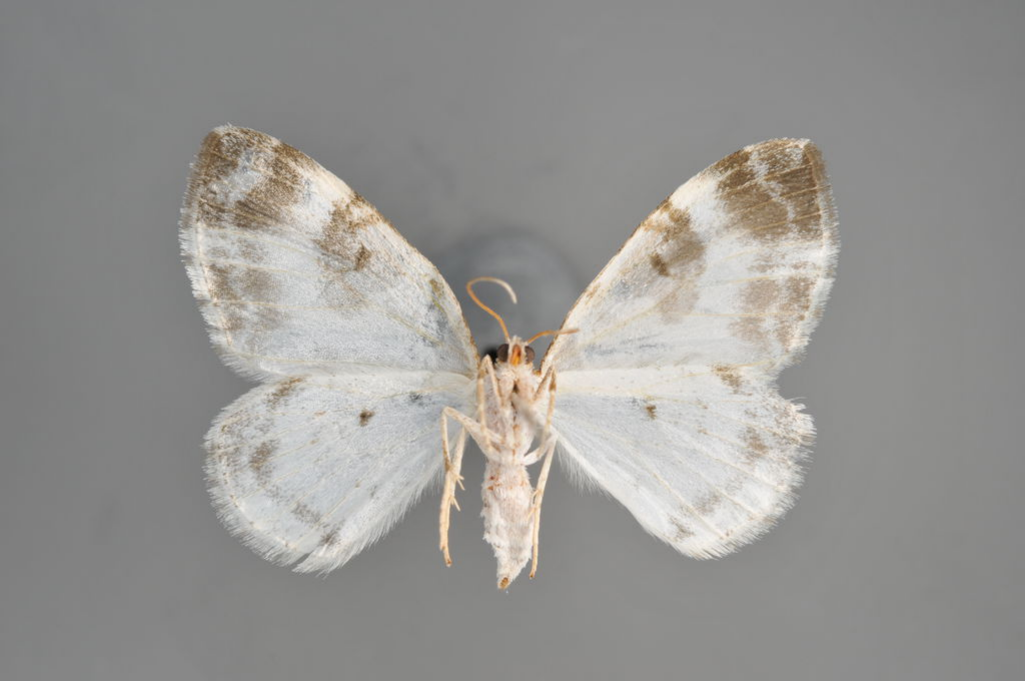
*Plemyria rubiginata*, underneath

**Figure 23.**
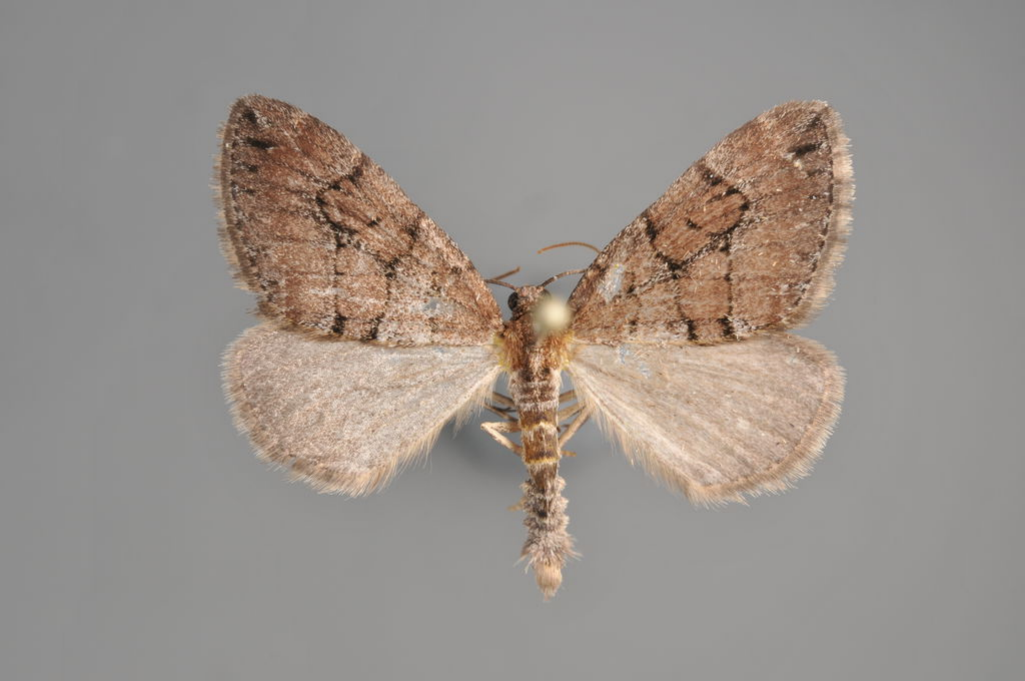
*Thera cognata*, above

**Figure 24.**
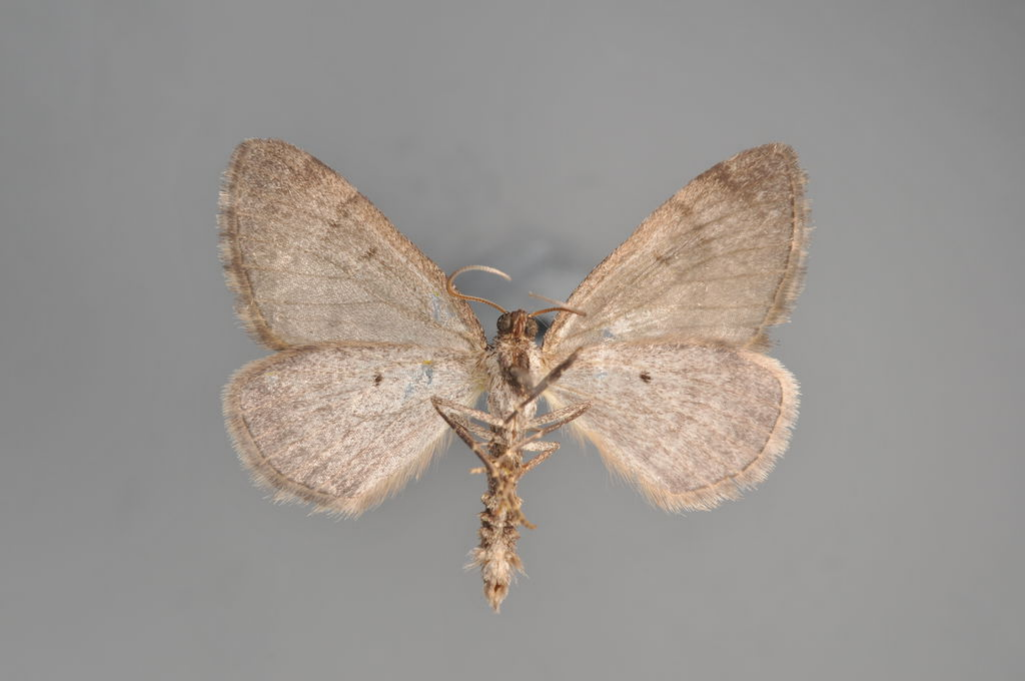
*Thera cognata*, underneath

**Figure 25.**
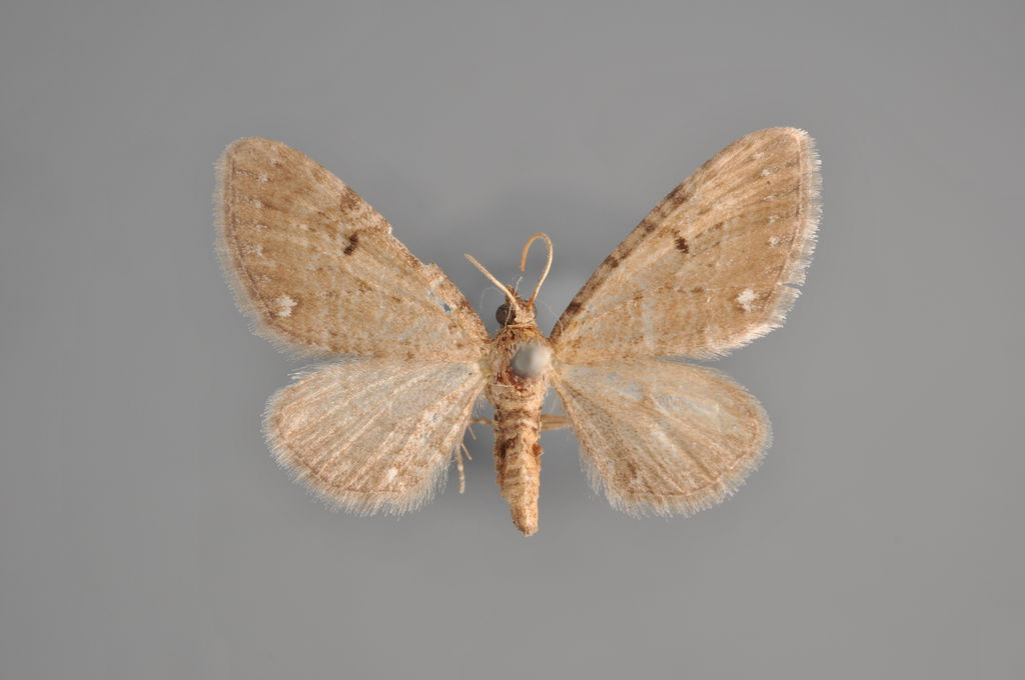
*Eupithecia absinthiata*, above

**Figure 26.**
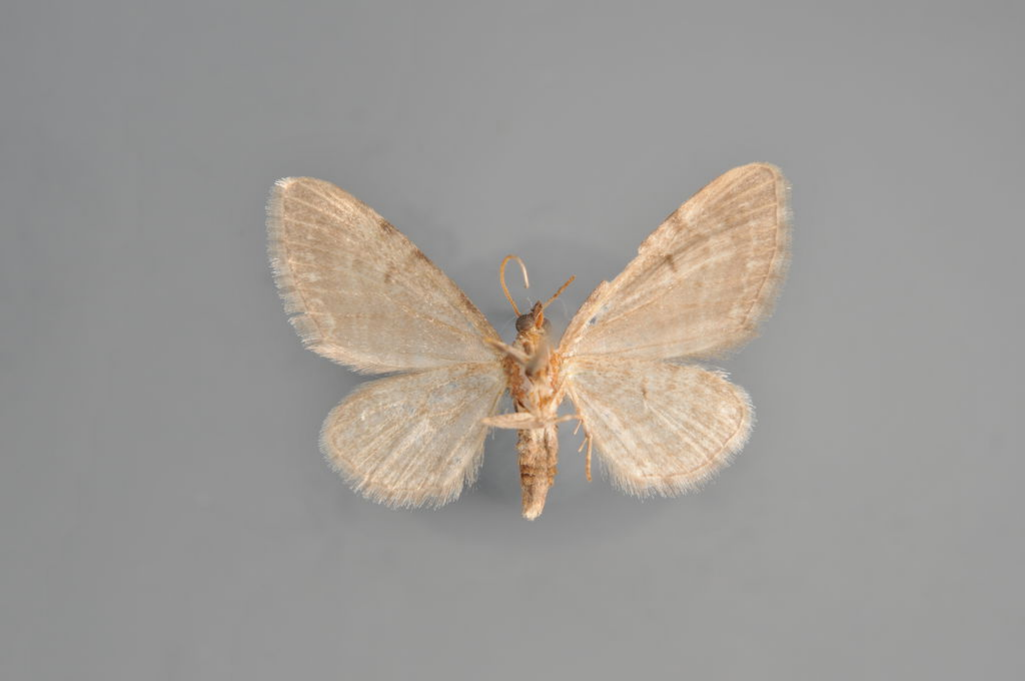
*Eupithecia absinthiata*, underneath

**Figure 27.**
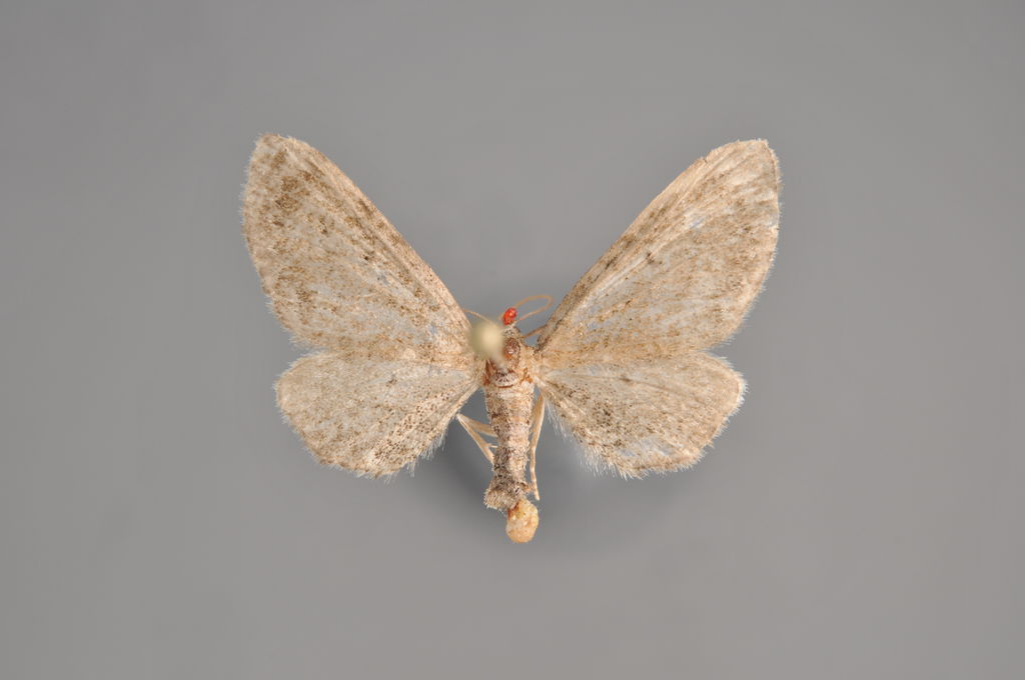
*Eupithecia denotata*, above

**Figure 28.**
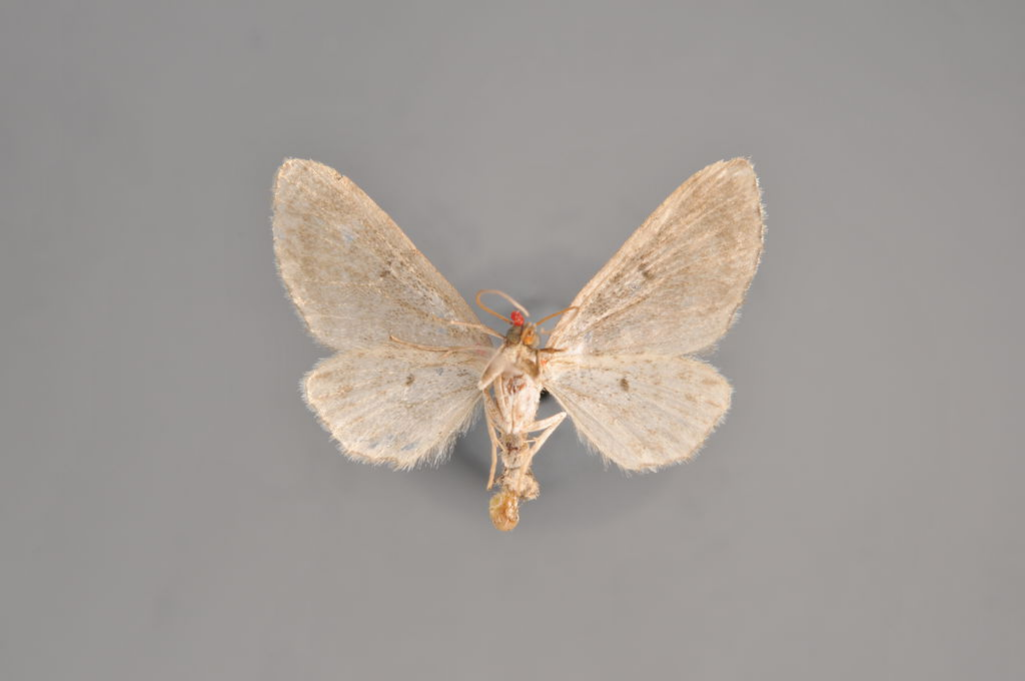
*Eupithecia denotata*, underneath

**Figure 29.**
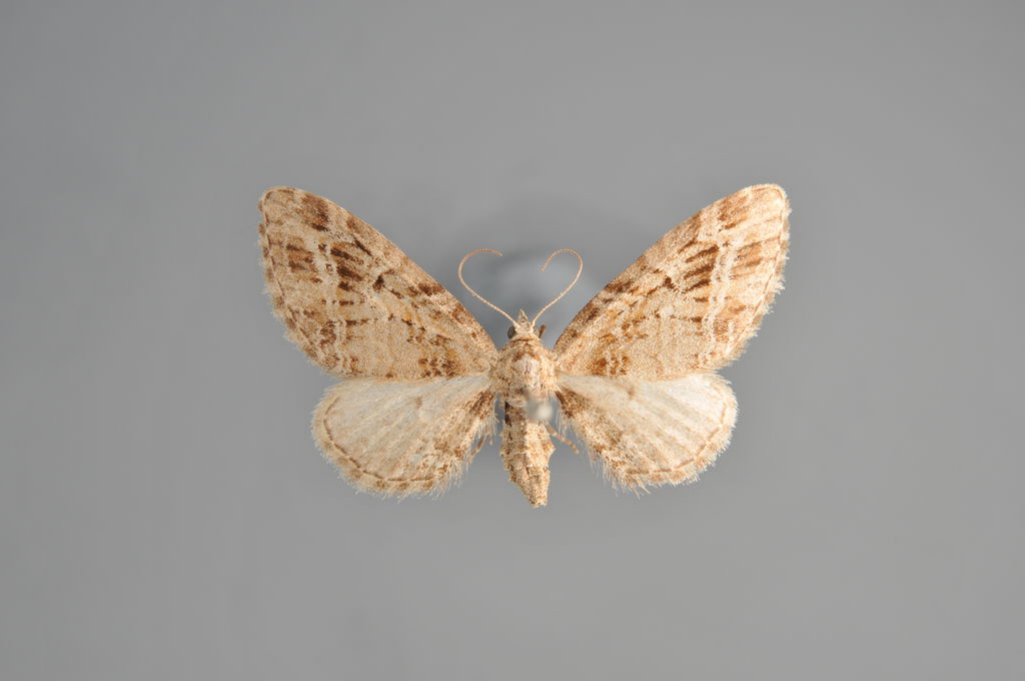
*Eupithecia exiguata*, above

**Figure 30.**
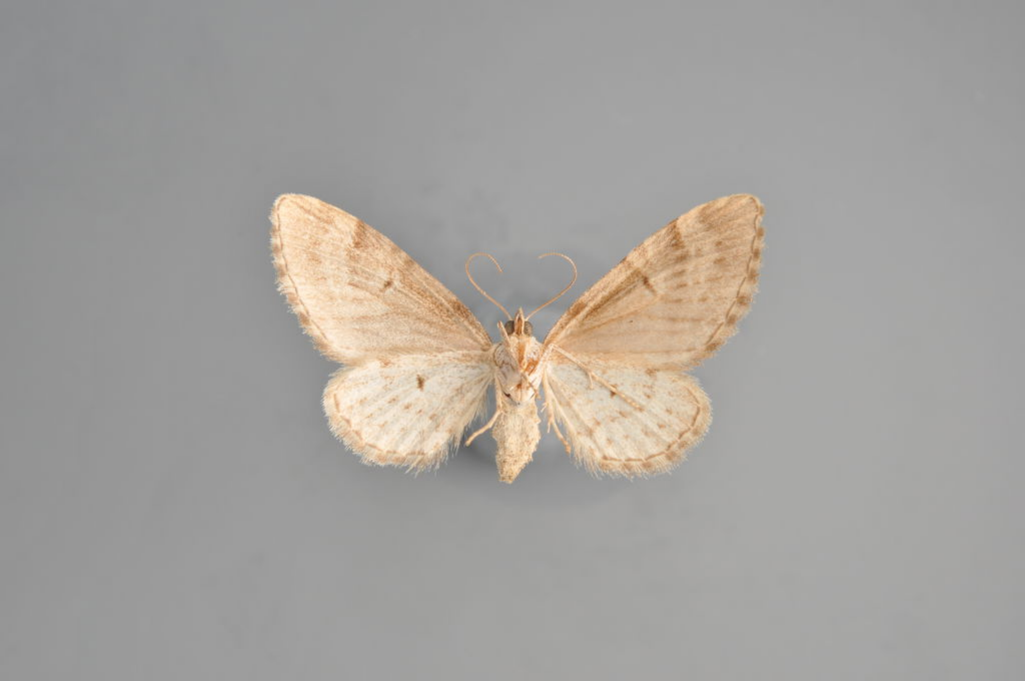
*Eupithecia exiguata*, underneath

**Figure 31.**
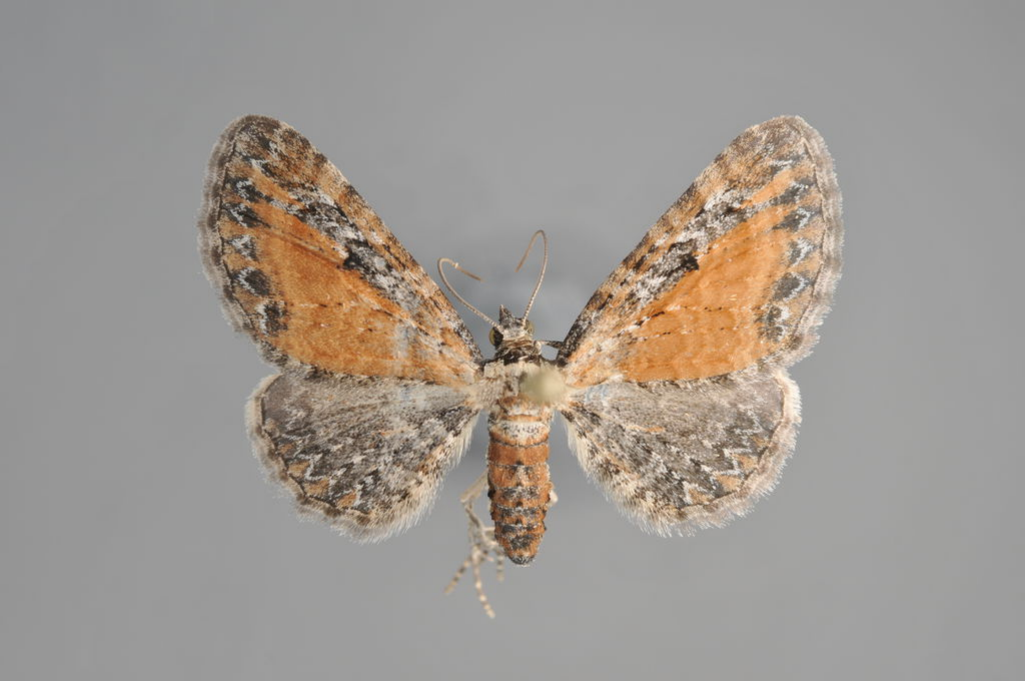
*Eupithecia icterata*, above

**Figure 32.**
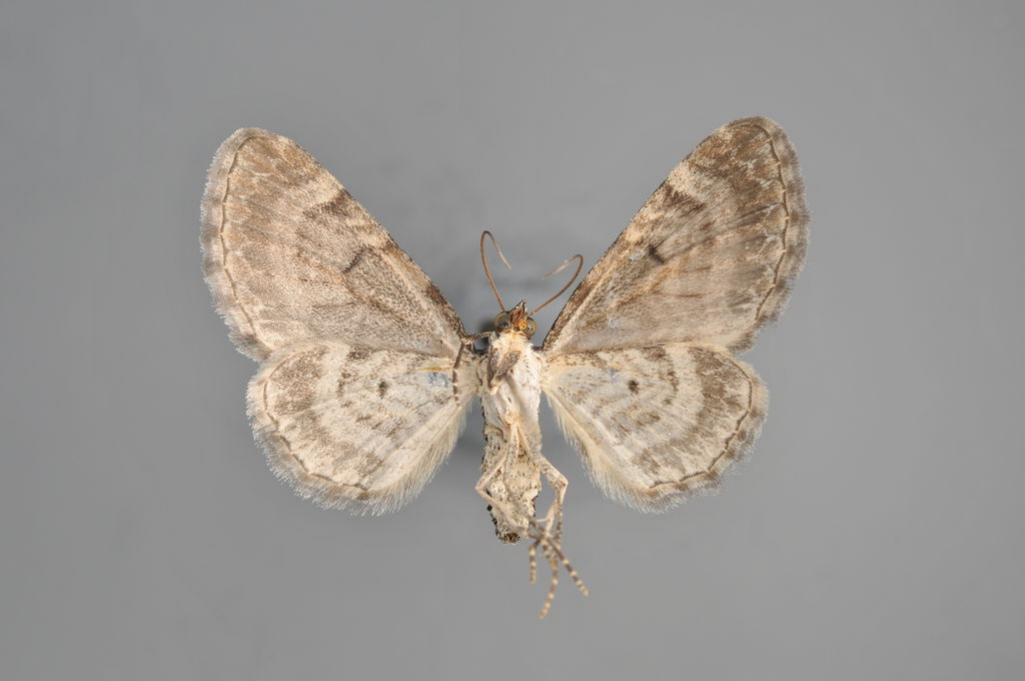
*Eupithecia icterata*, underneath

**Figure 33.**
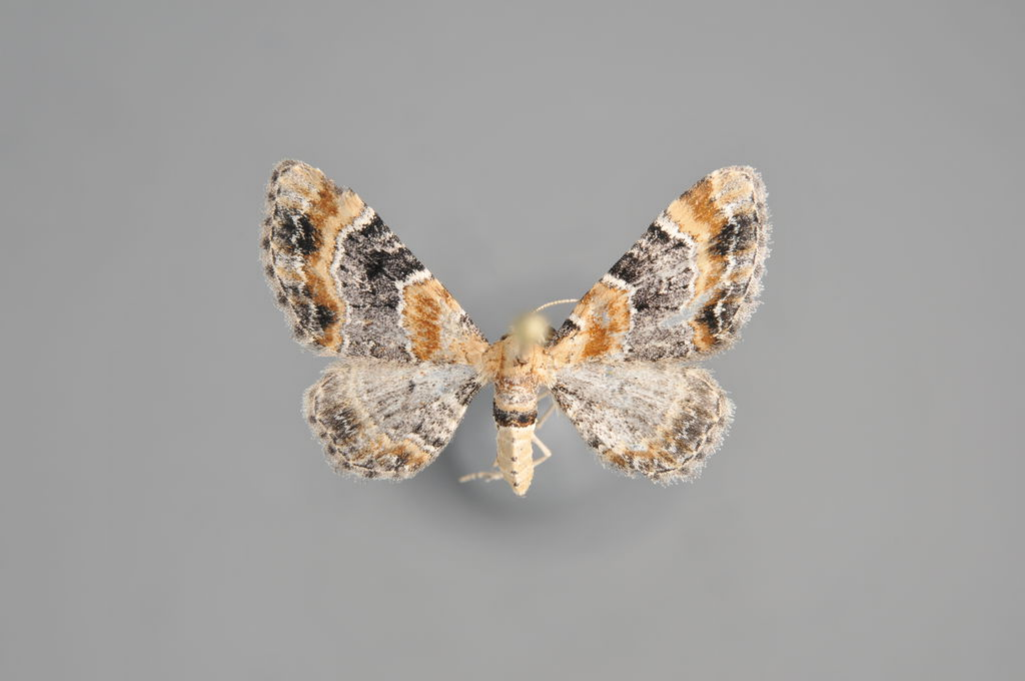
*Eupithecia linariata*, above

**Figure 34.**
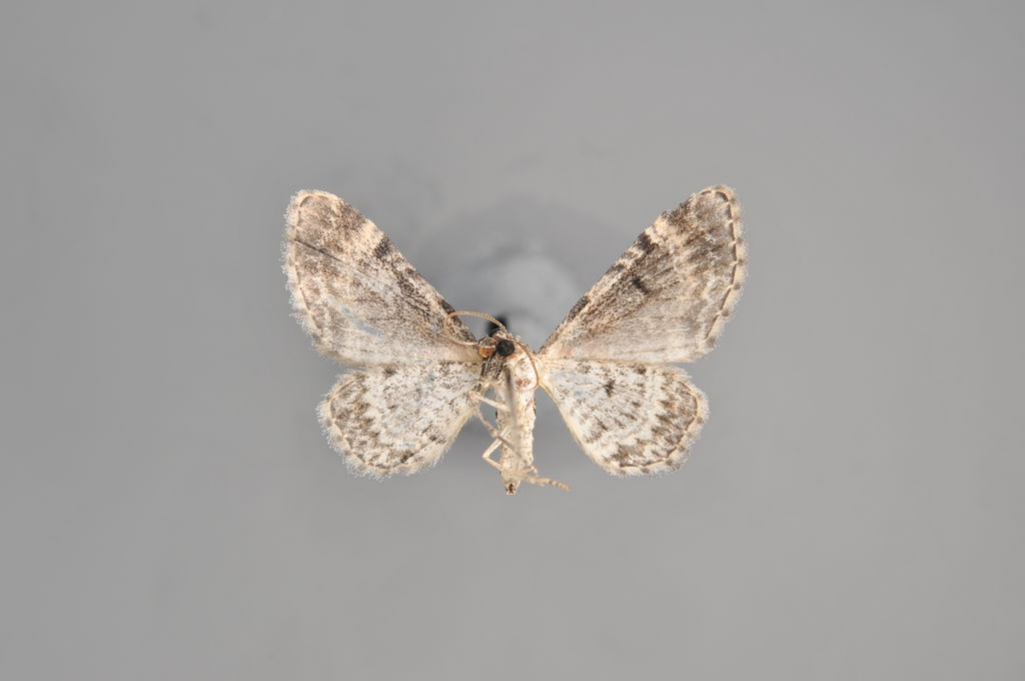
*Eupithecia linariata*, underneath

**Figure 35.**
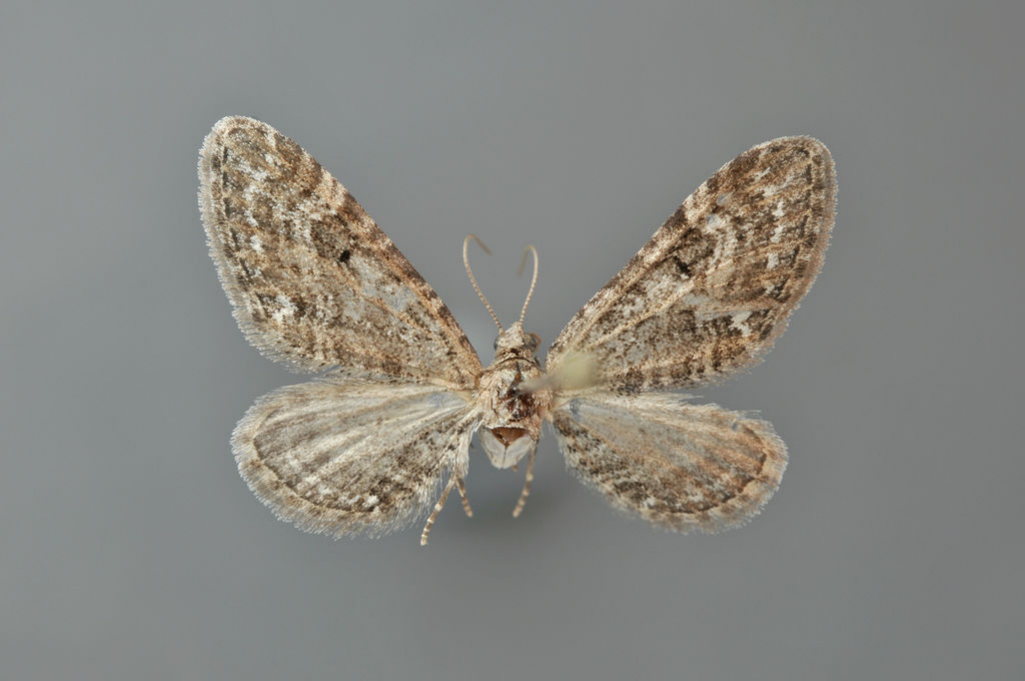
*Eupithecia nanata*, above, abdomen missing

**Figure 36.**
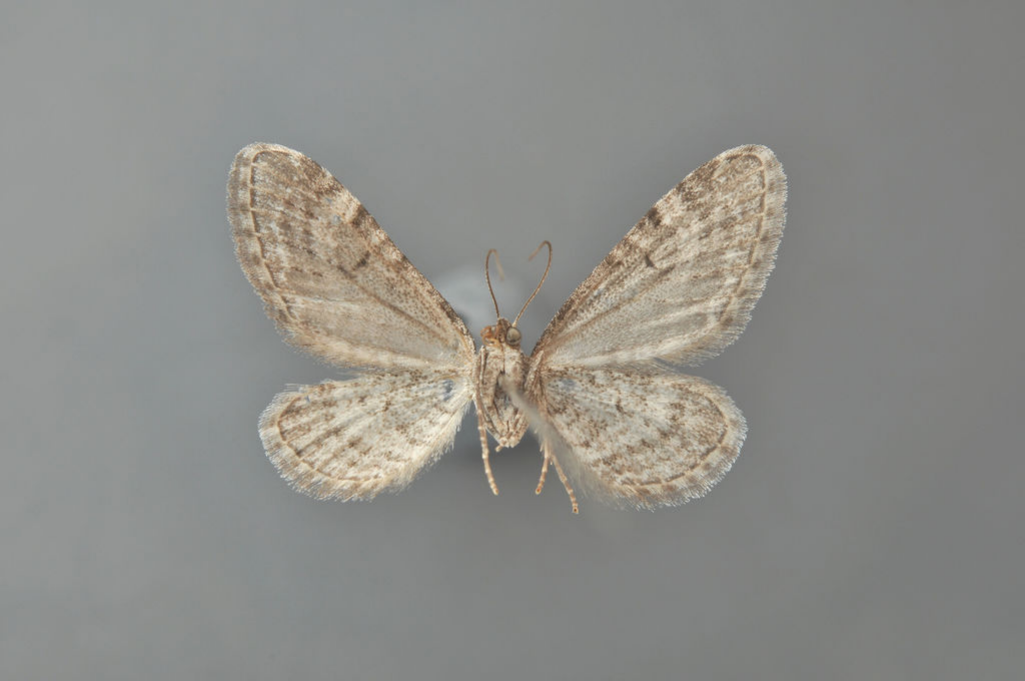
*Eupithecia nanata*, underneath, abdomen missing

**Figure 37.**
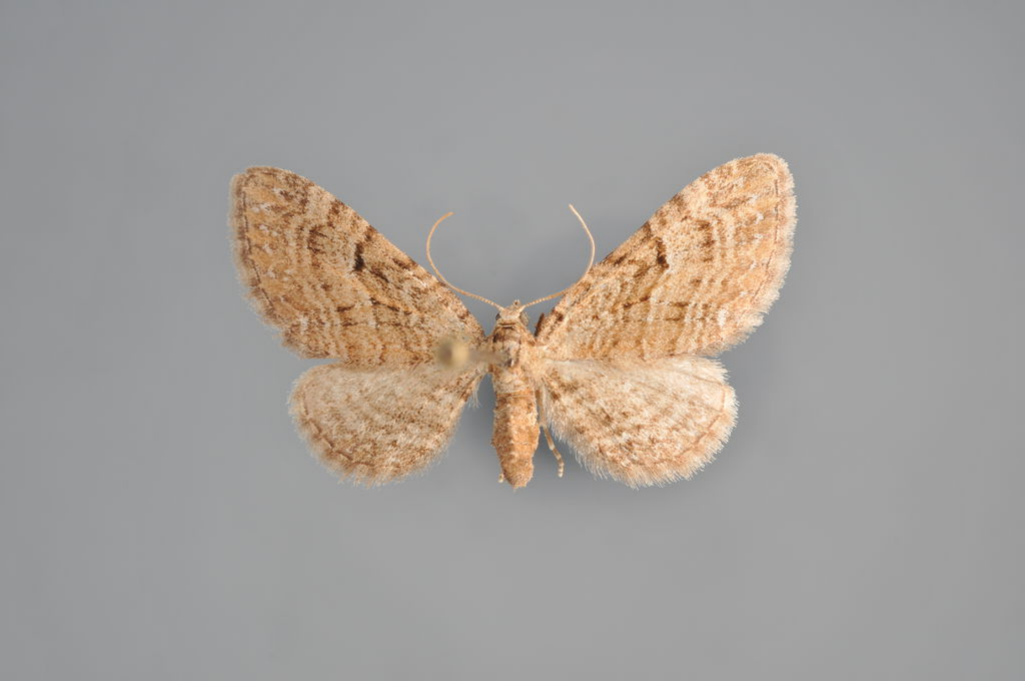
*Eupithecia pusillata*, above

**Figure 38.**
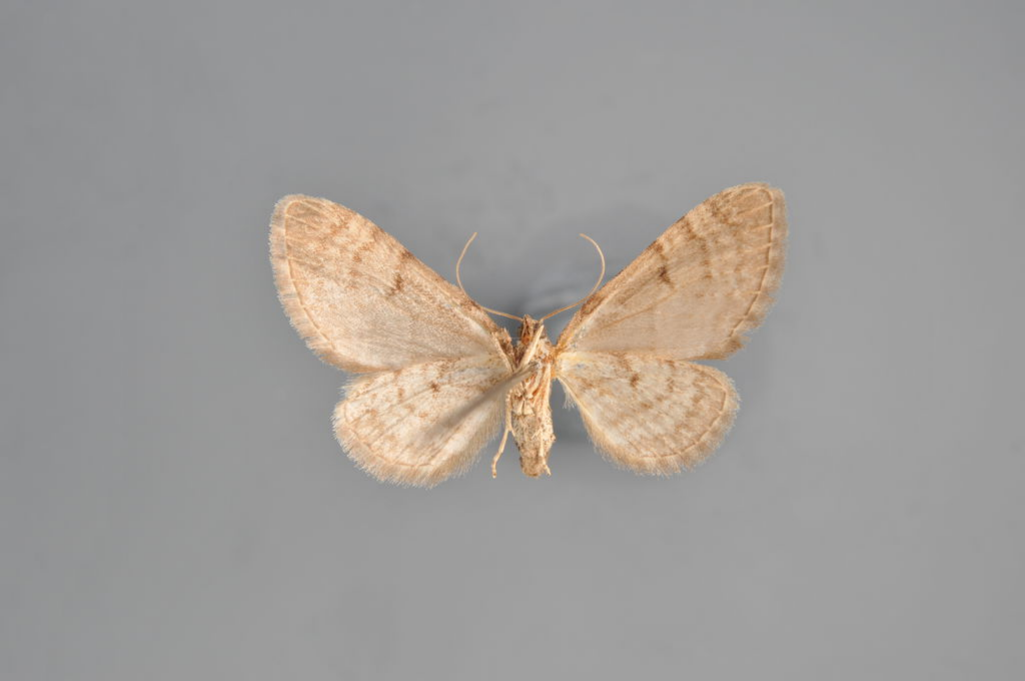
*Eupithecia pusillata*, underneath

**Figure 39.**
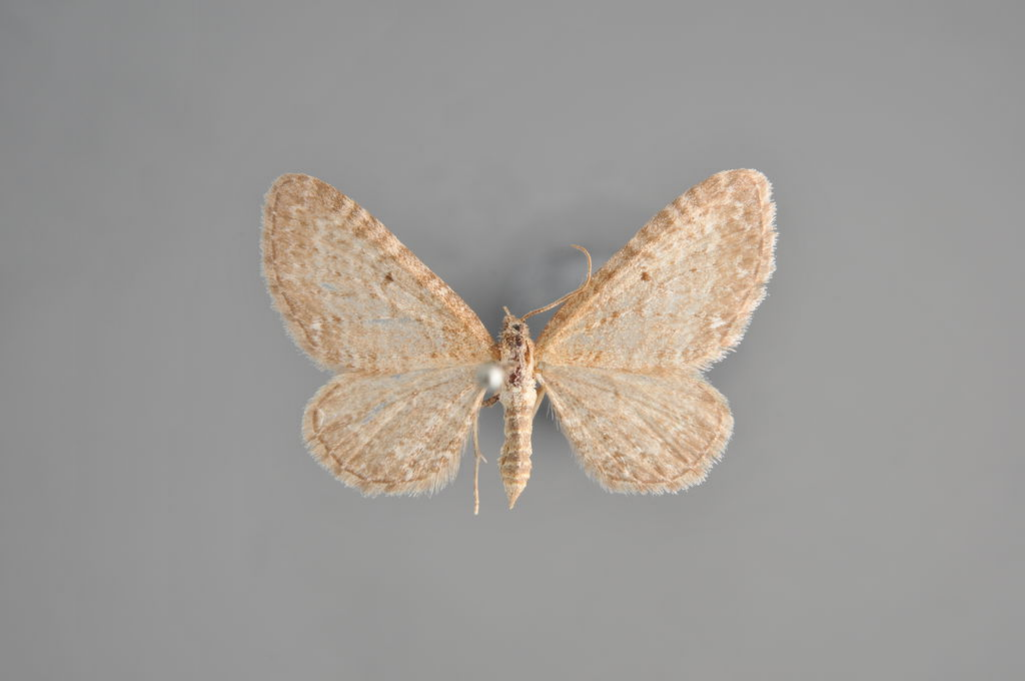
*Eupithecia satyrata*, above

**Figure 40.**
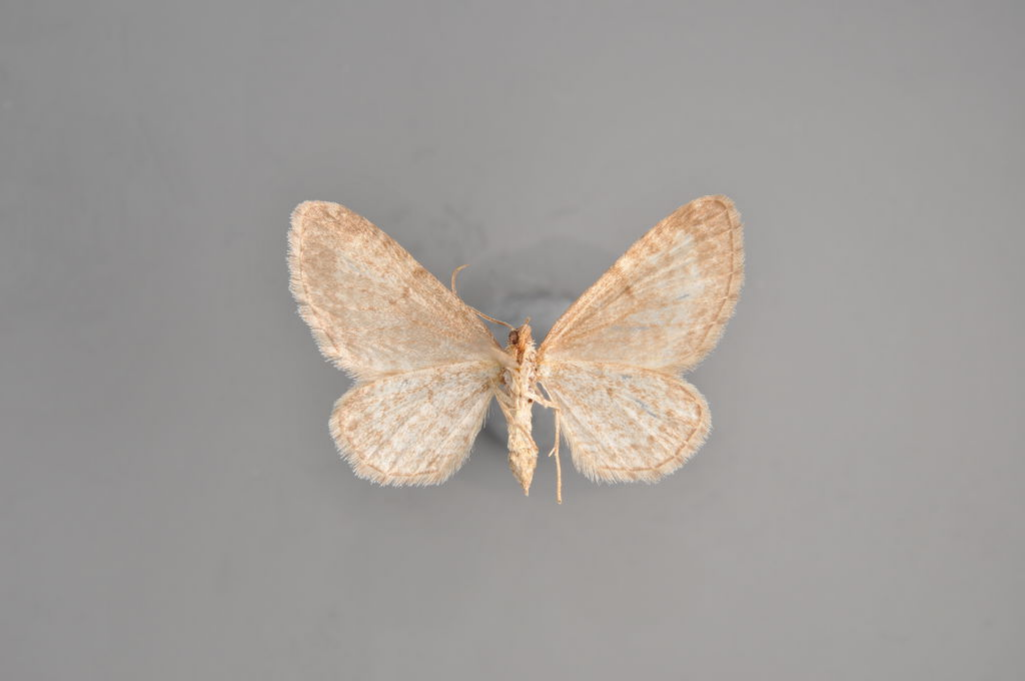
*Eupithecia satyrata*, underneath

**Figure 41.**
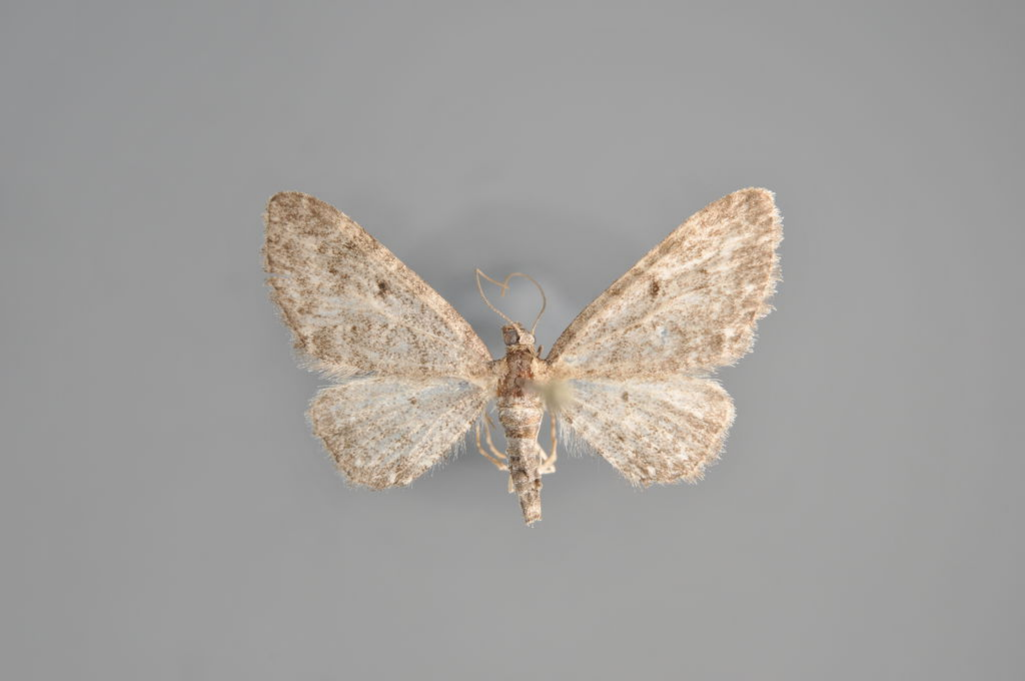
*Eupithecia subfuscata*, above

**Figure 42.**
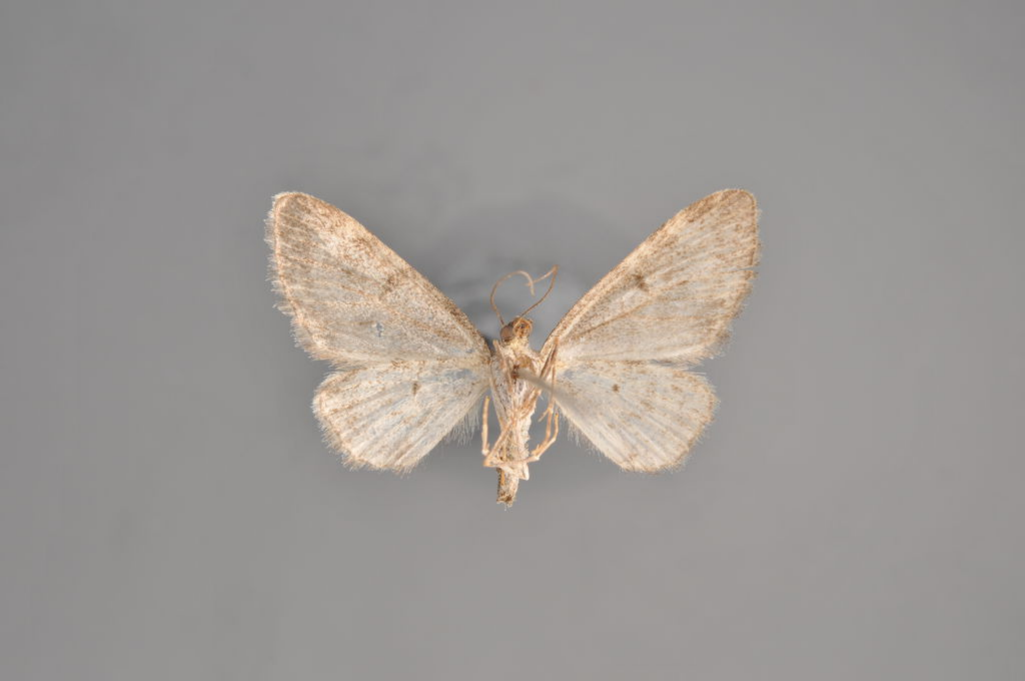
*Eupithecia subfuscata*, underneath

**Figure 43.**
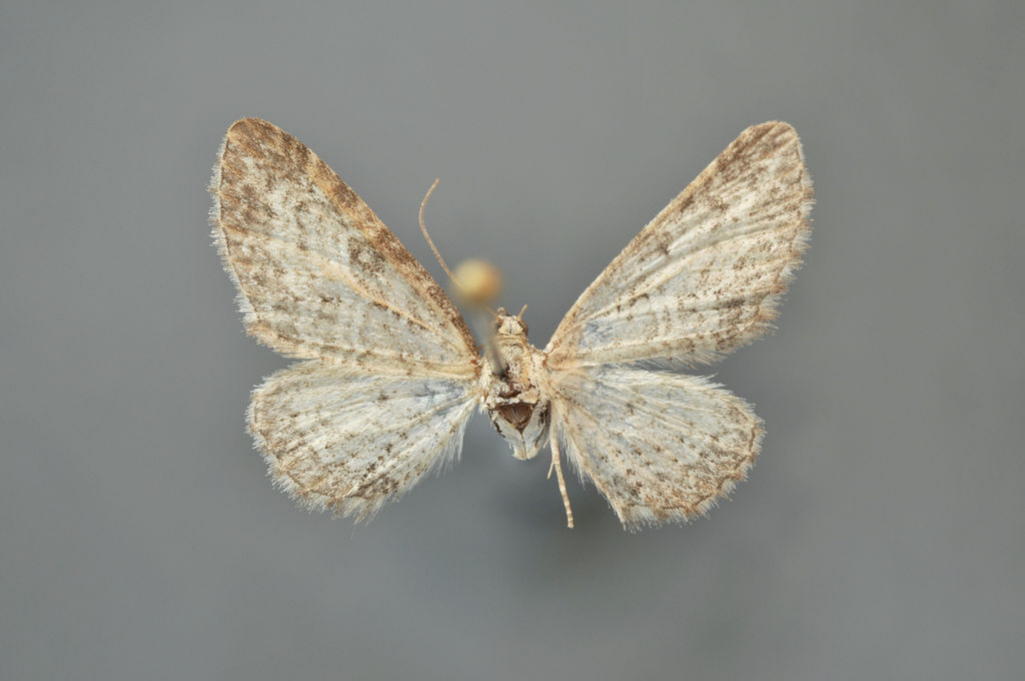
*Eupithecia subumbrata*, above, abdomen missing

**Figure 44.**
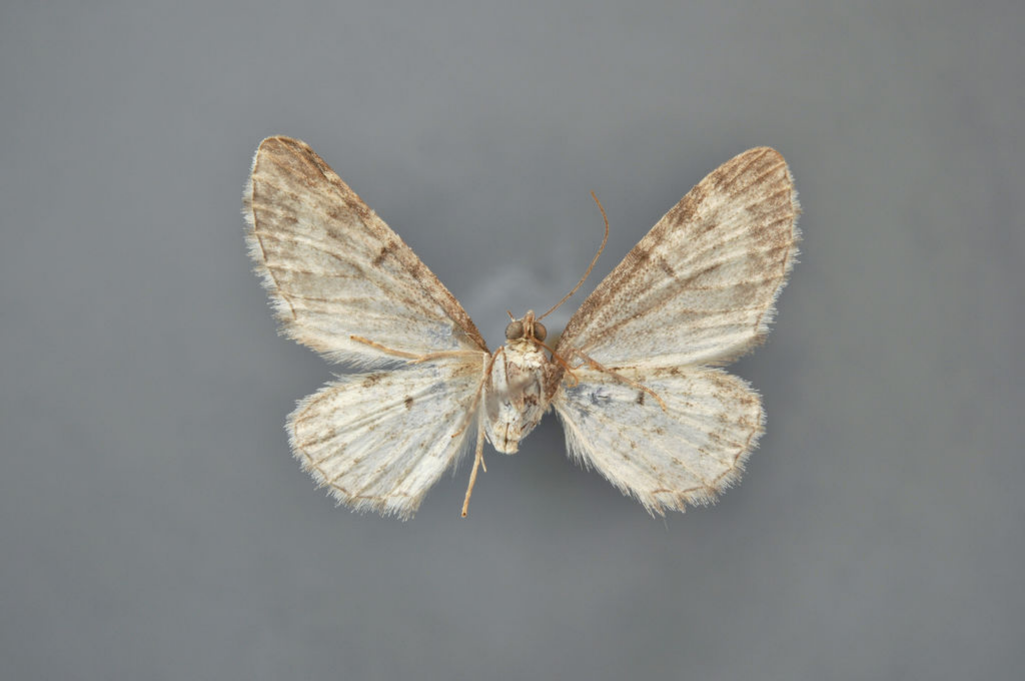
*Eupithecia subumbrata*, underneath, abdomen missing

**Figure 45.**
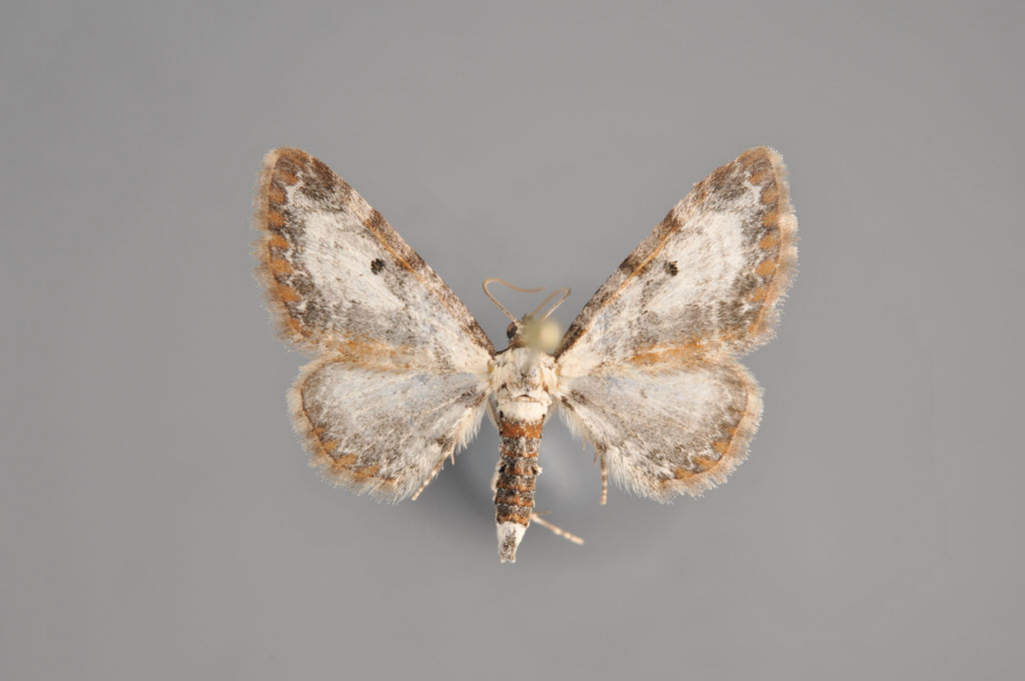
*Eupithecia succenturiata*, above

**Figure 46.**
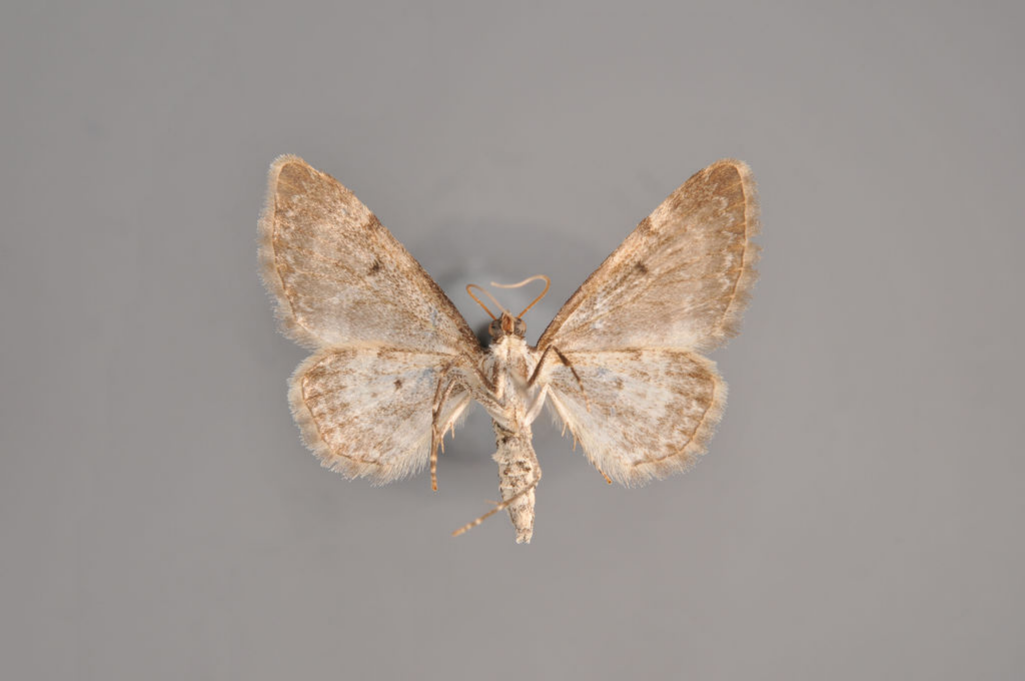
*Eupithecia succenturiata*, underneath

**Figure 47.**
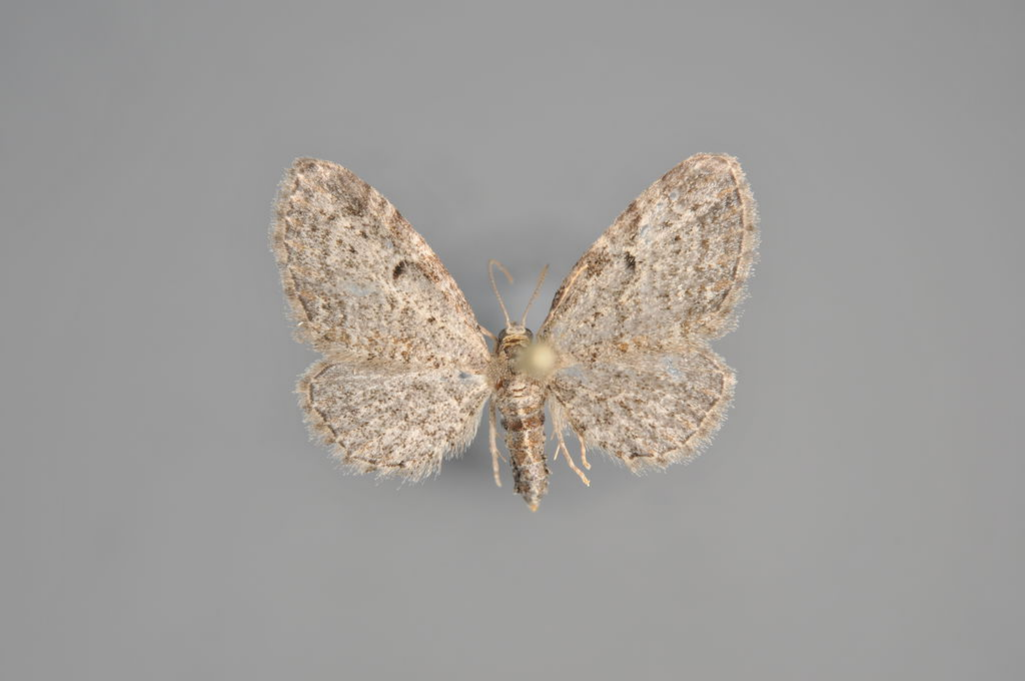
*Eupithecia tenuiata*, above

**Figure 48.**
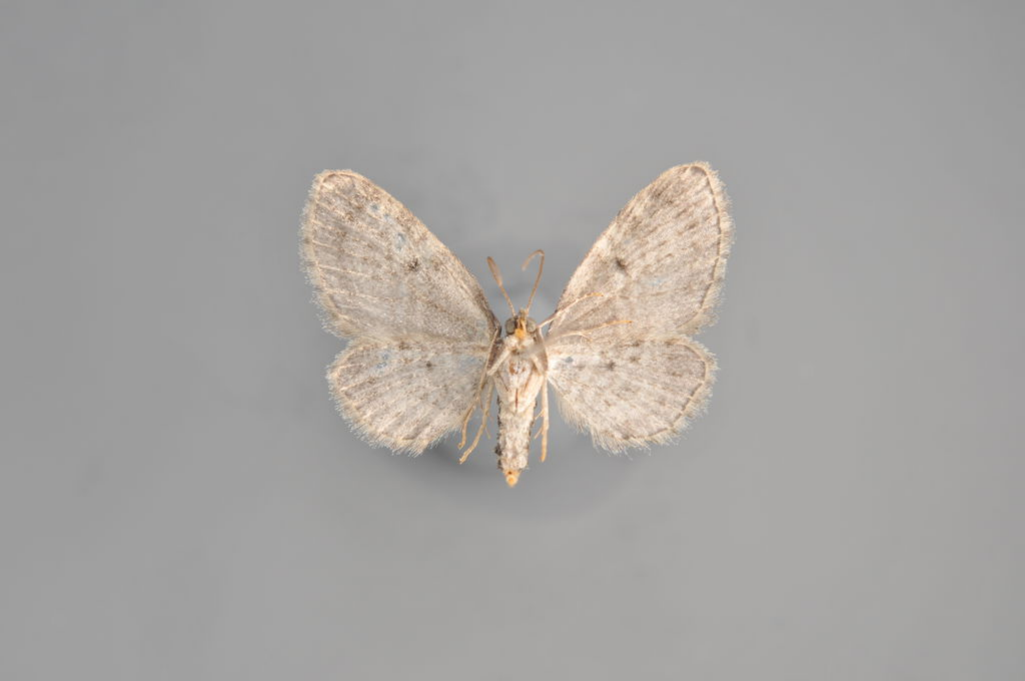
*Eupithecia tenuiata*, underneath

**Figure 49.**
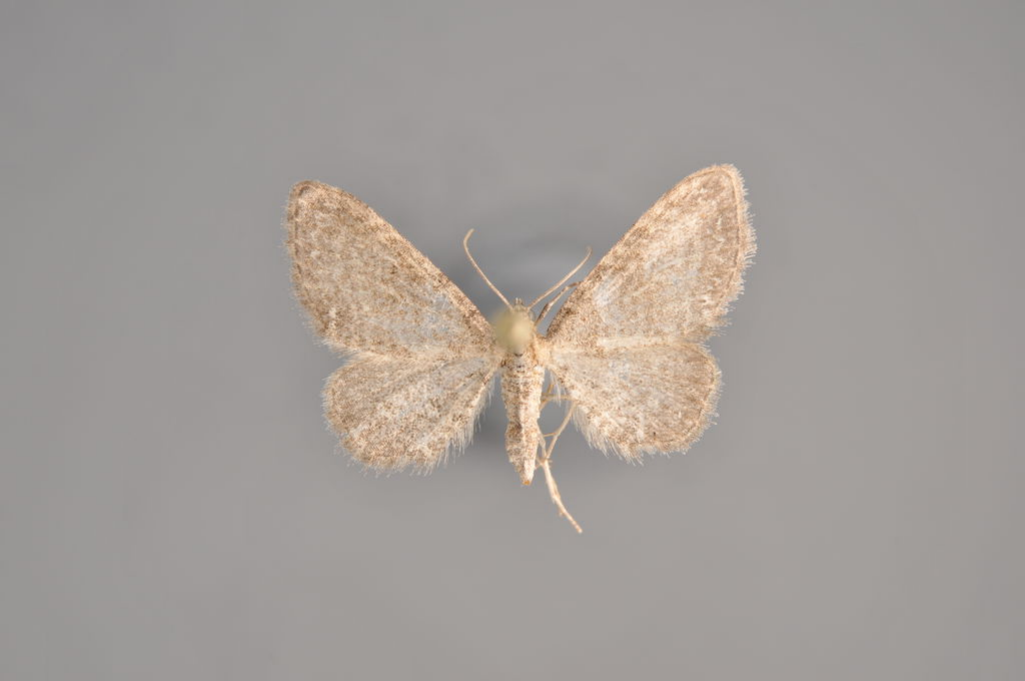
*Eupithecia valerianata*, above

**Figure 50.**
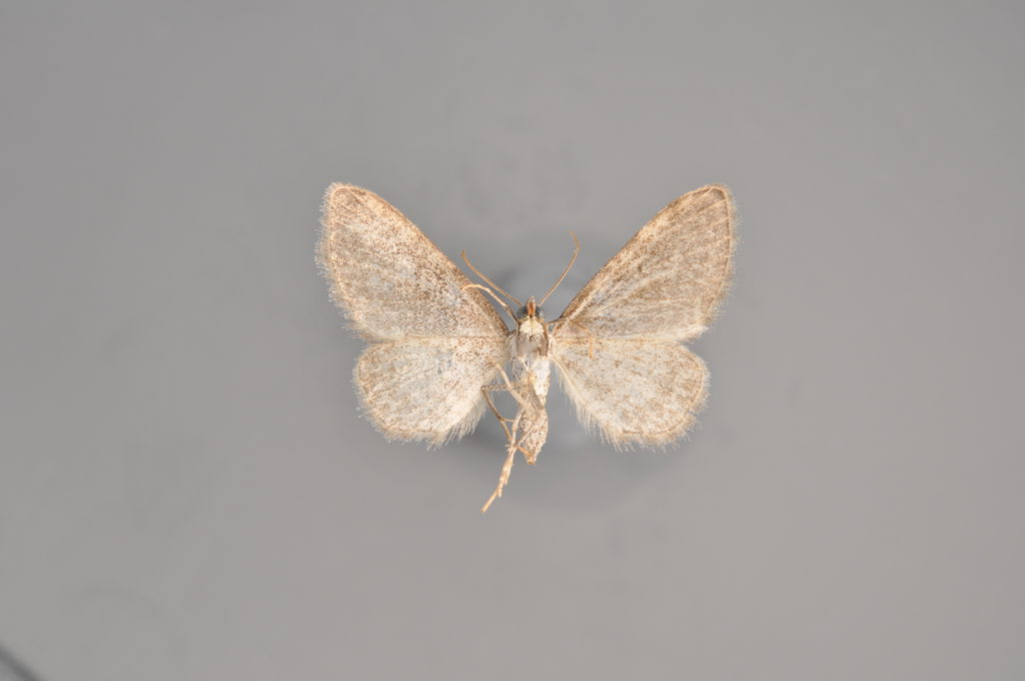
*Eupithecia valerianata*, underneath

**Figure 51.**
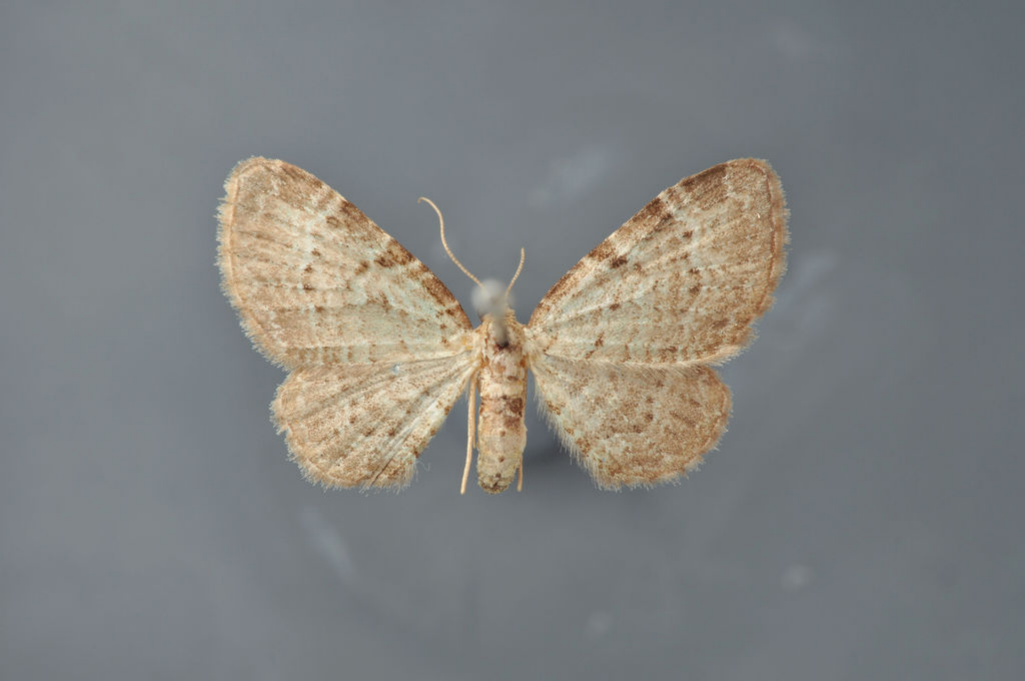
*Pasiphila chloerata*, above

**Figure 52.**
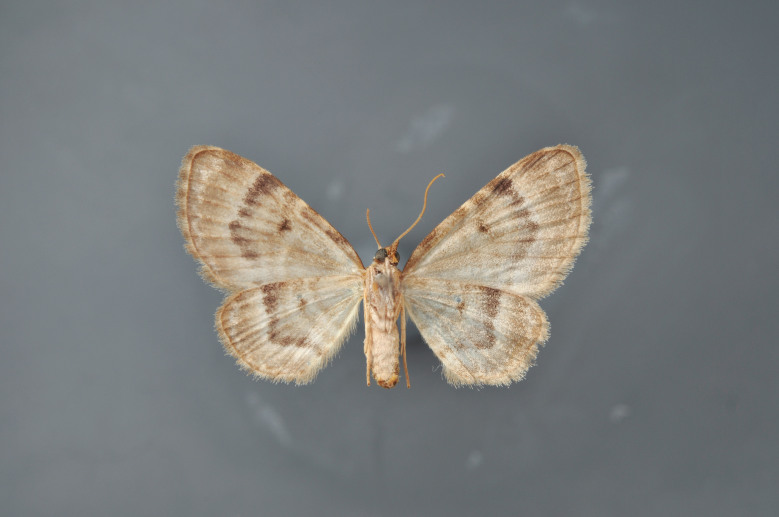
*Pasiphila chloerata*, underneath

**Figure 53.**
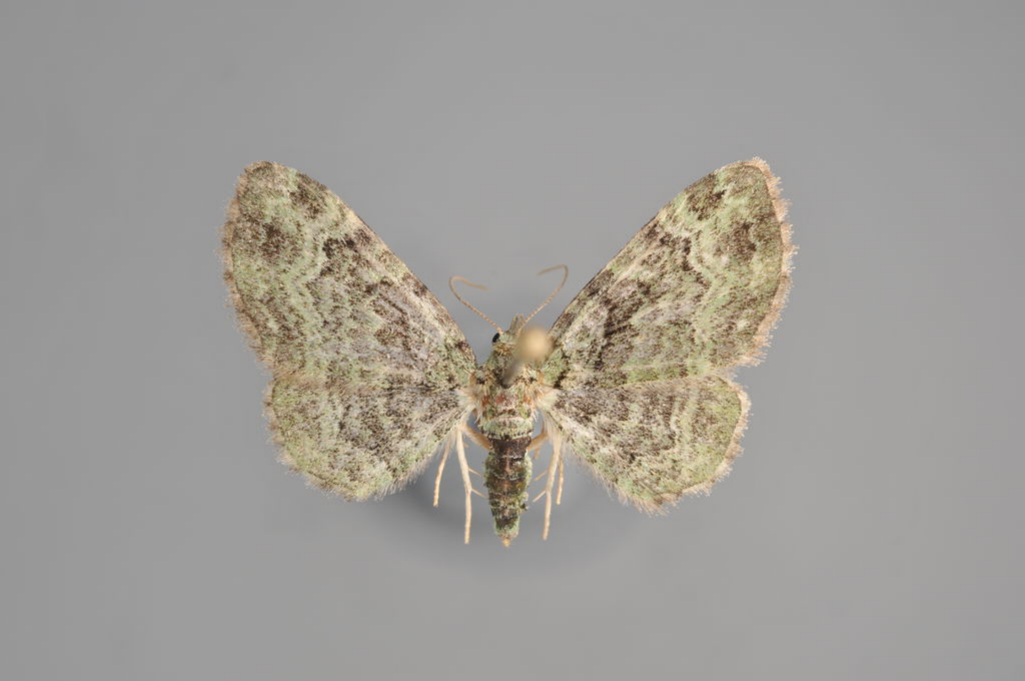
*Pasiphila rectangulata*, above

**Figure 54.**
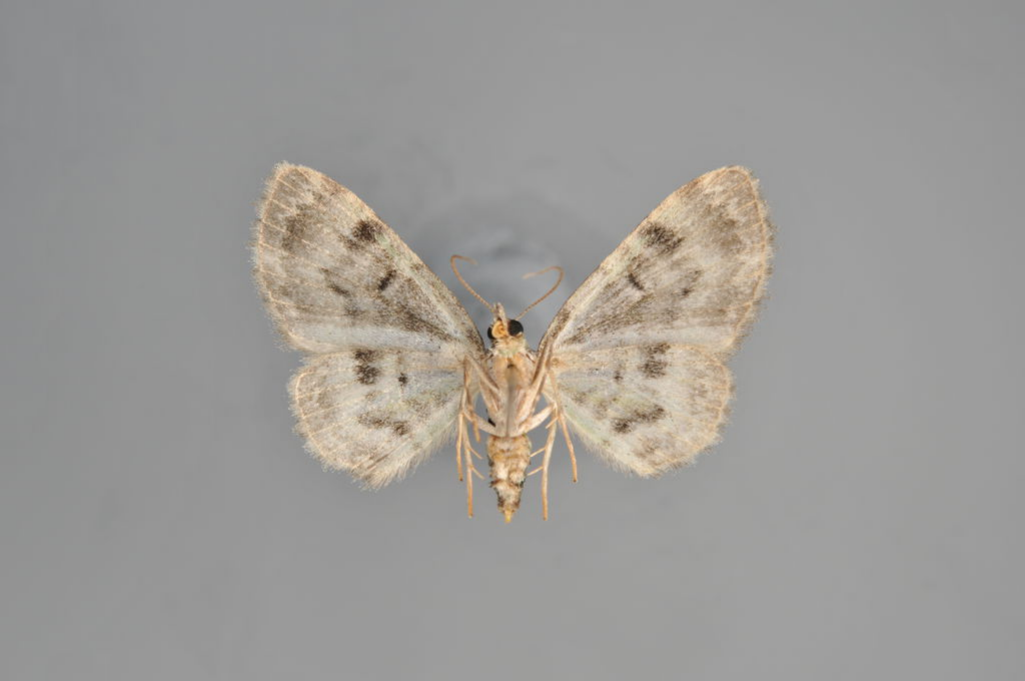
*Pasiphila rectangulata*, underneath

**Figure 55.**
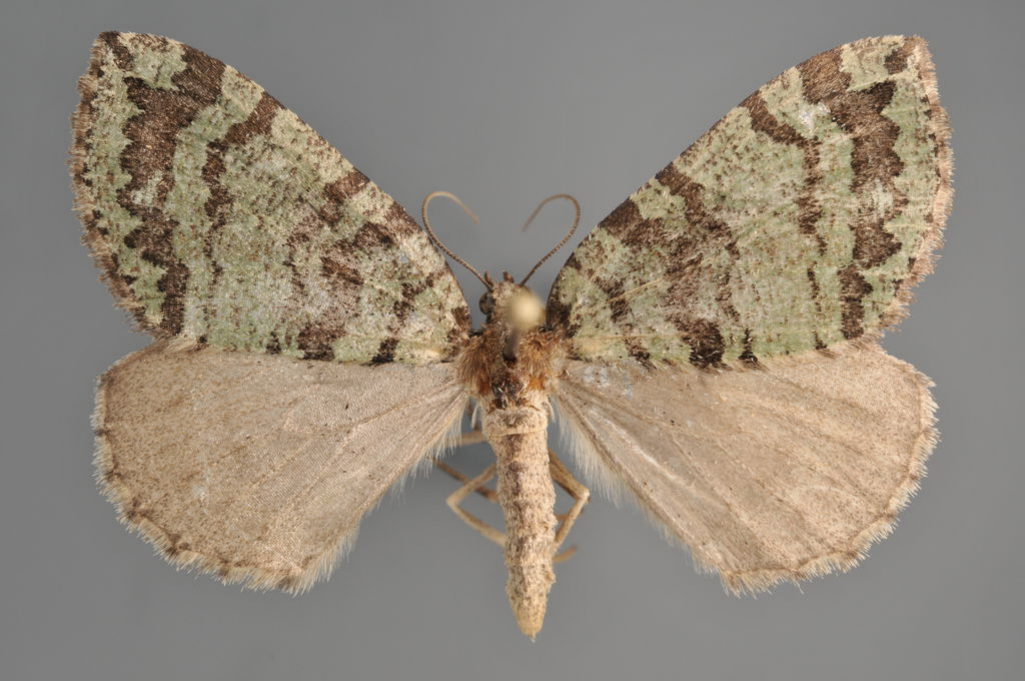
*Hydriomena furcata*, above

**Figure 56.**
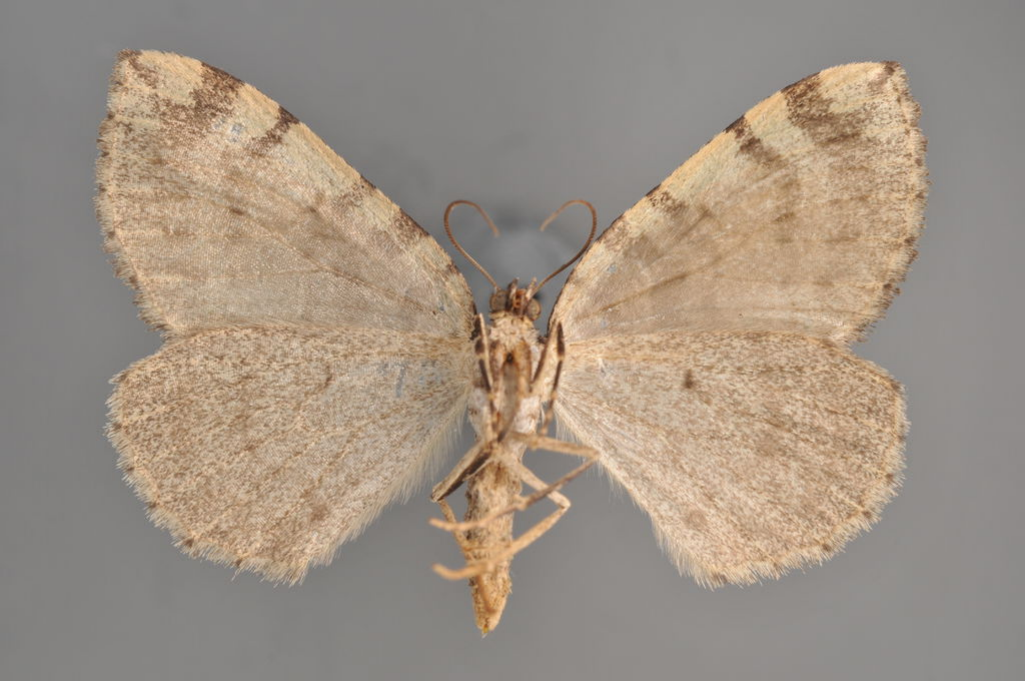
*Hydriomena furcata*, under

**Figure 57.**
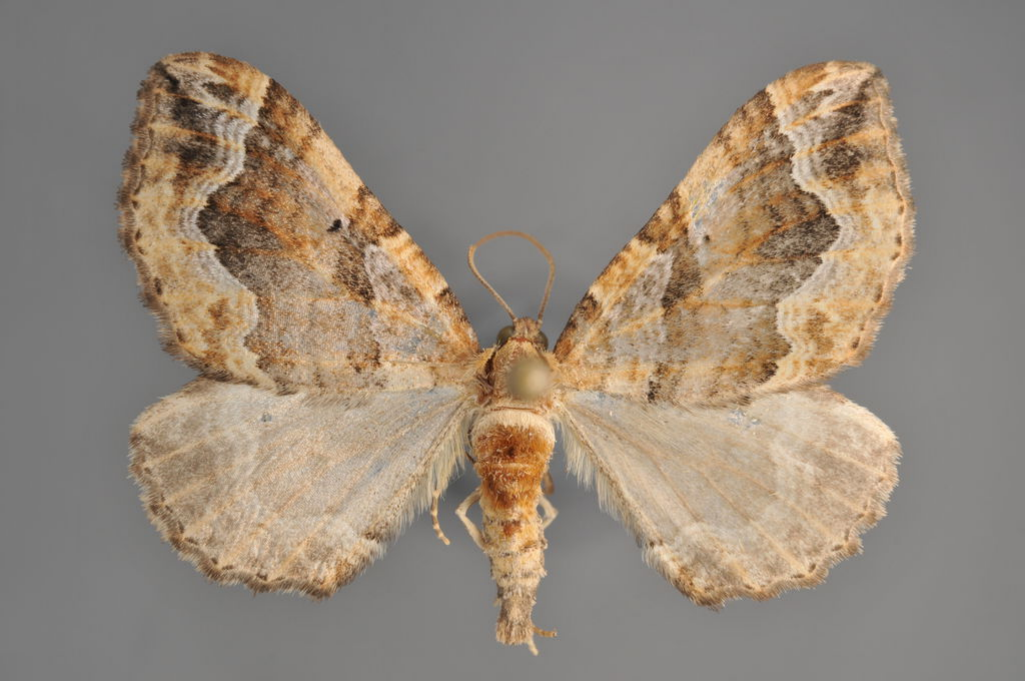
*Pelurga comitata*, above

**Figure 58.**
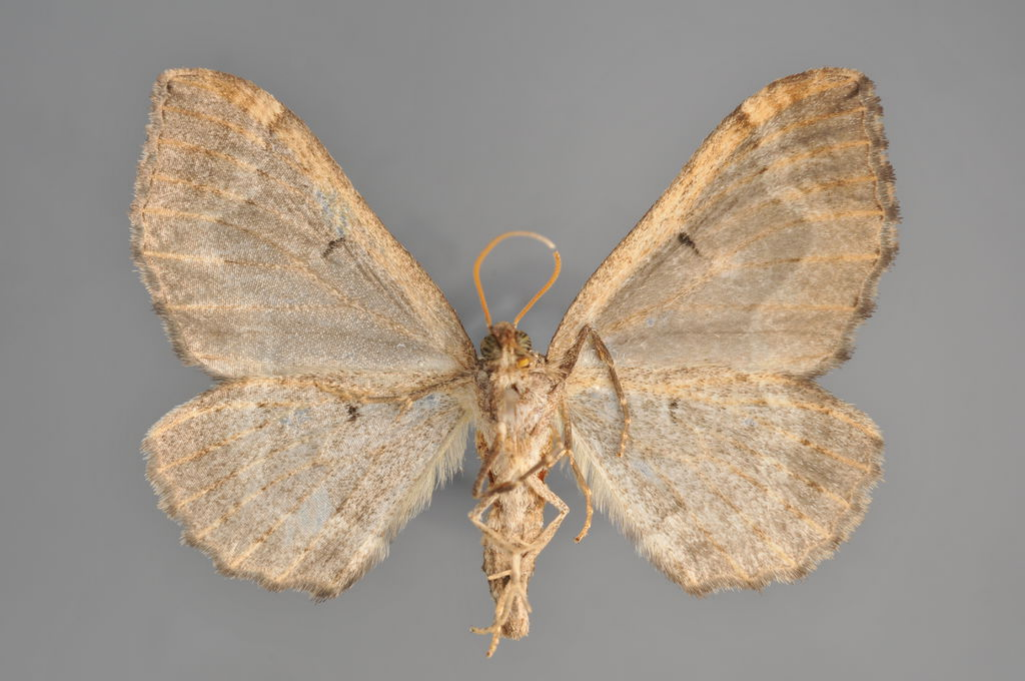
*Pelurga comitata*, underneath

**Figure 59.**
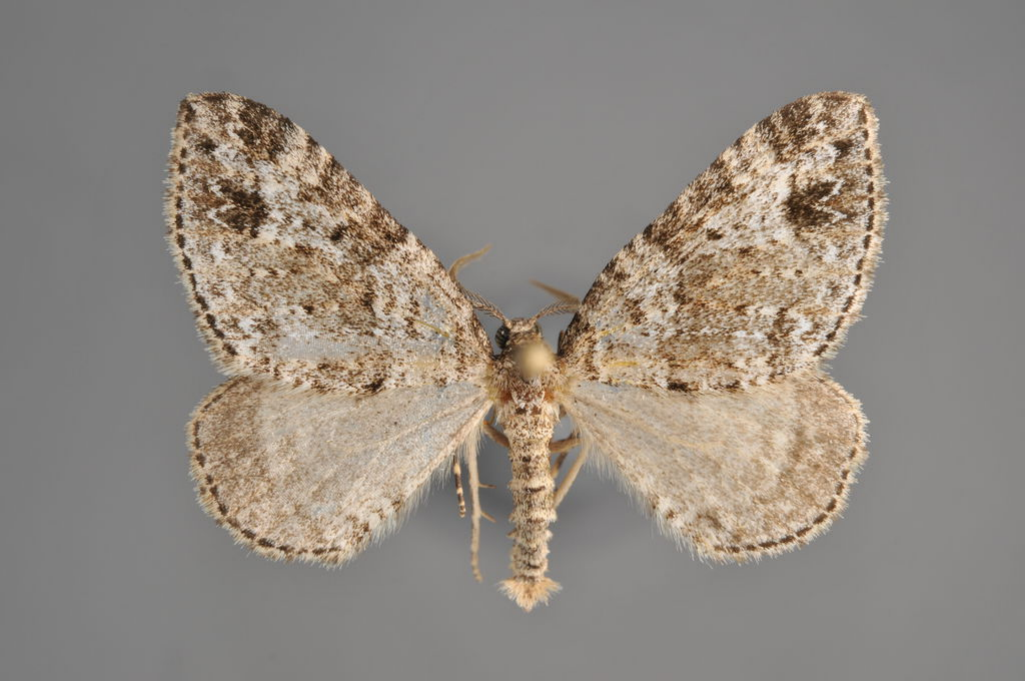
*Mesotype didymata*, above

**Figure 60.**
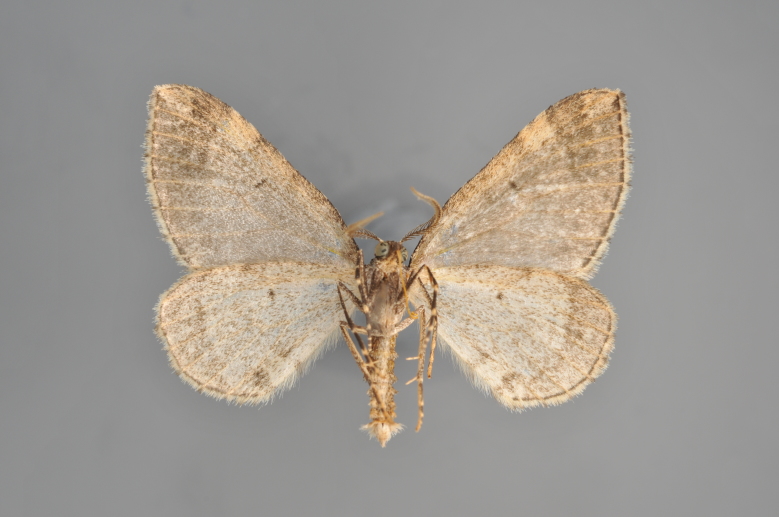
*Mesotype didymata*, underneath

**Figure 61.**
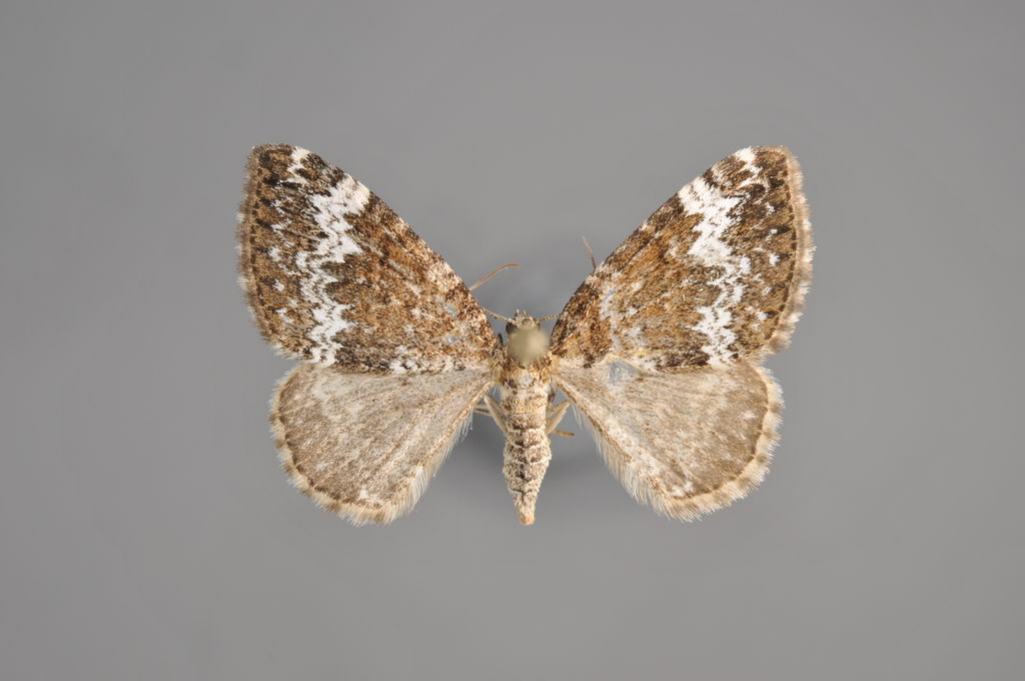
*Perizoma alchemillata*, above

**Figure 62.**
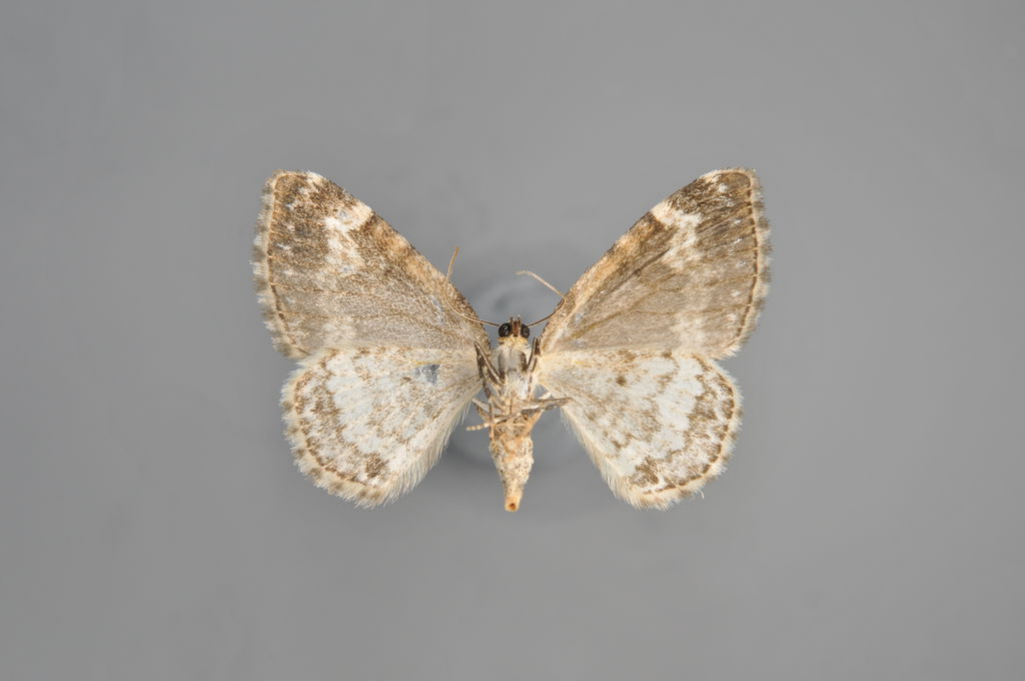
*Perizoma alchemillata*, underneath

**Figure 63.**
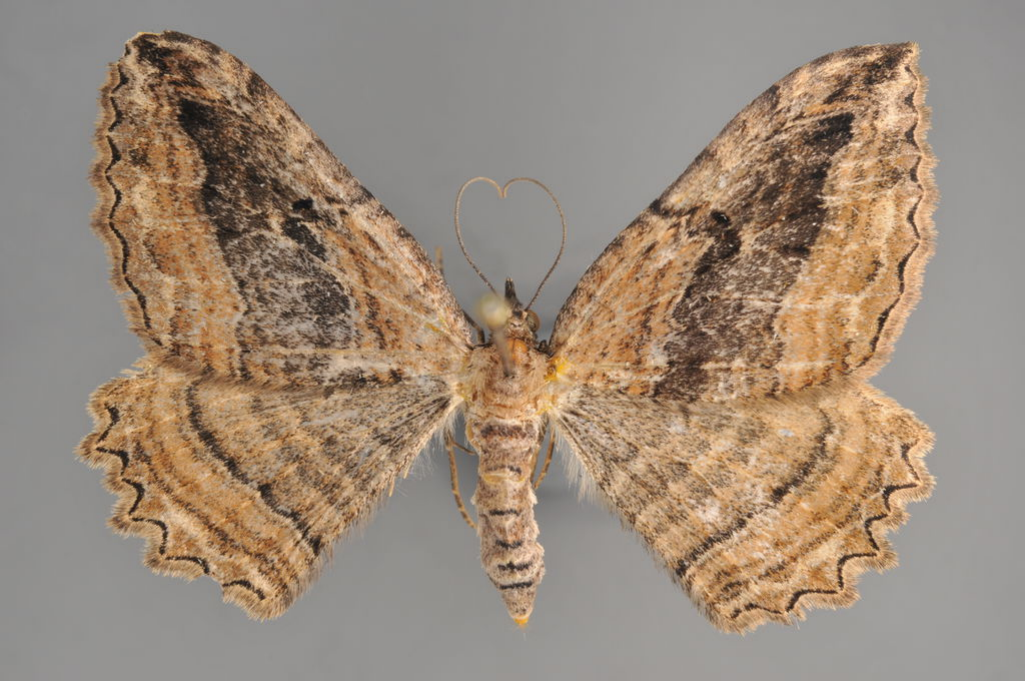
*Philereme transversata*, above

**Figure 64.**
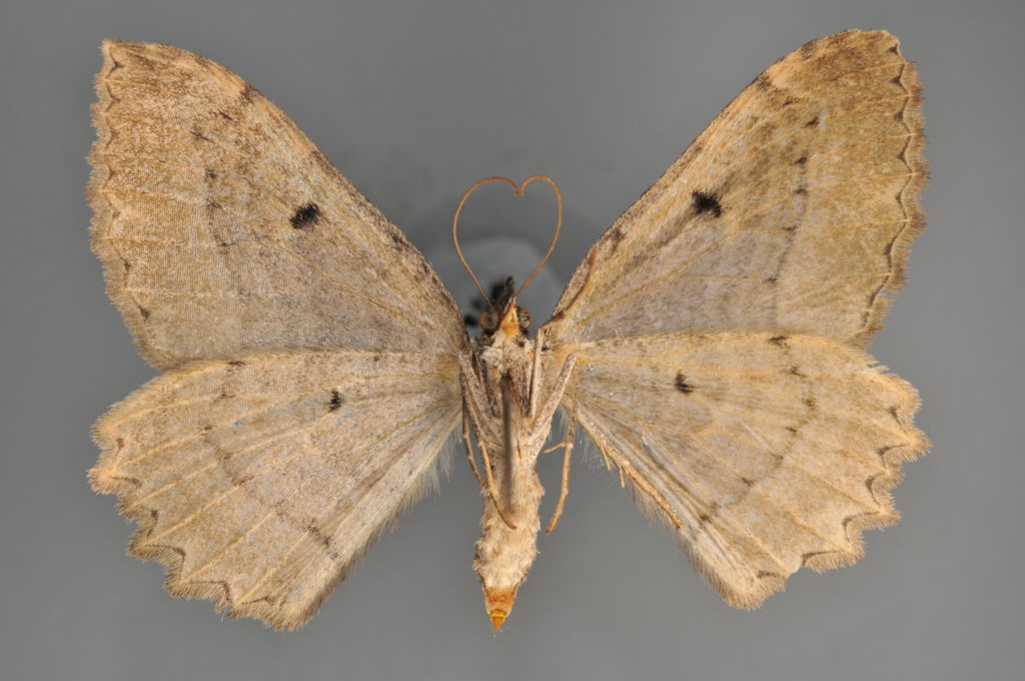
*Philereme transversata*, underneath

**Figure 65.**
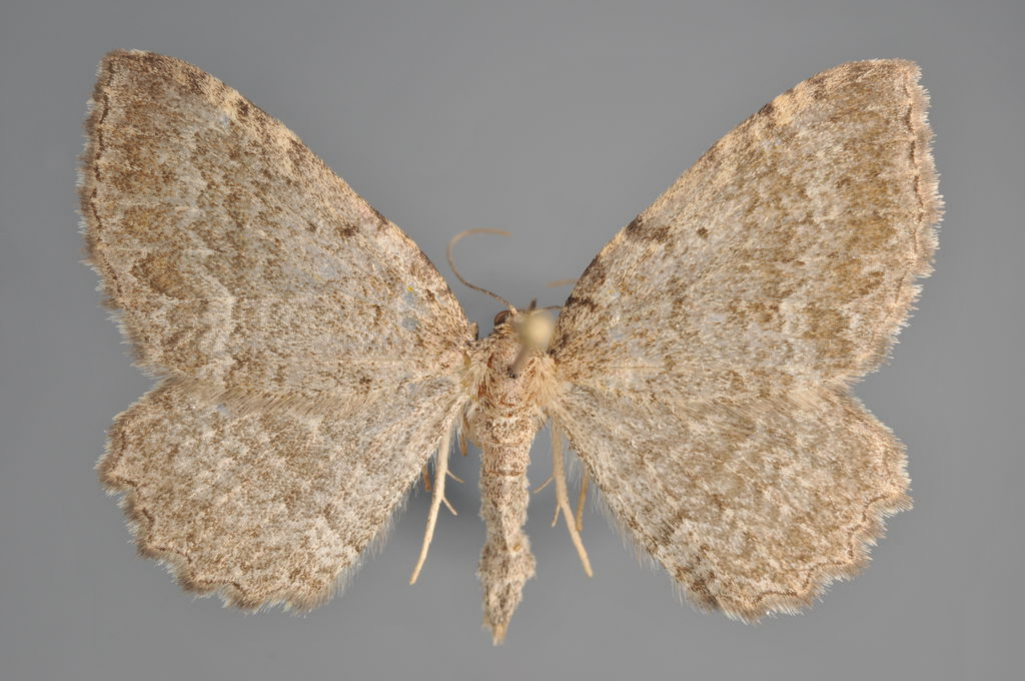
*Philereme vetulata*, above

**Figure 66.**
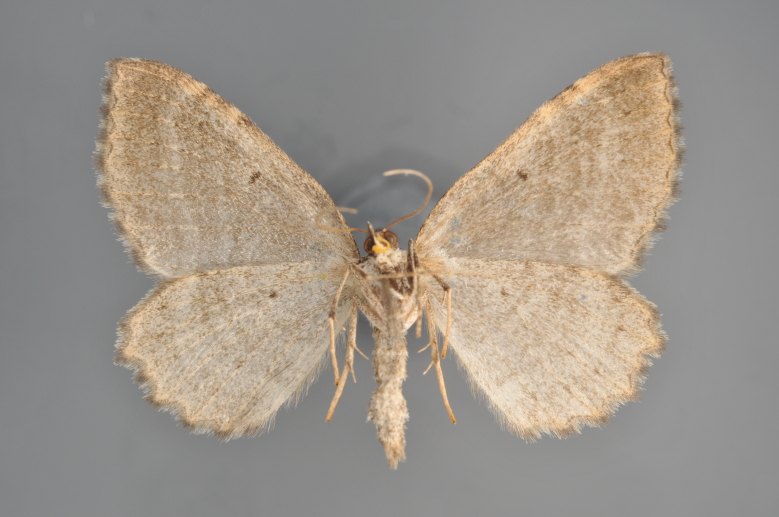
*Philereme vetulata*, underneath

**Figure 67.**
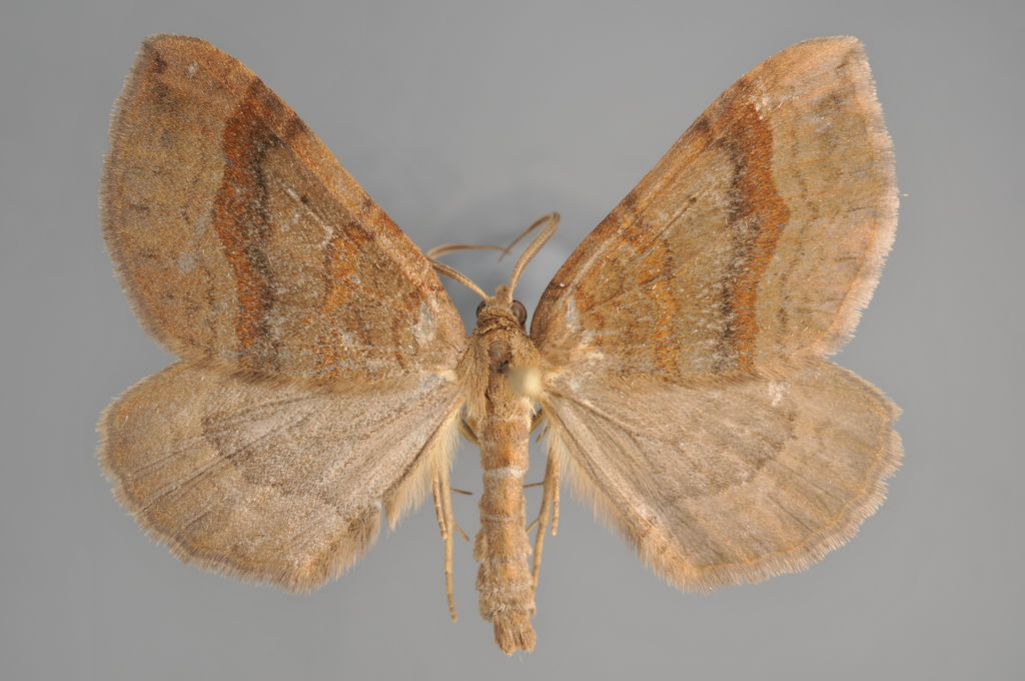
*Scotopteryx chenopodiata*, above

**Figure 68.**
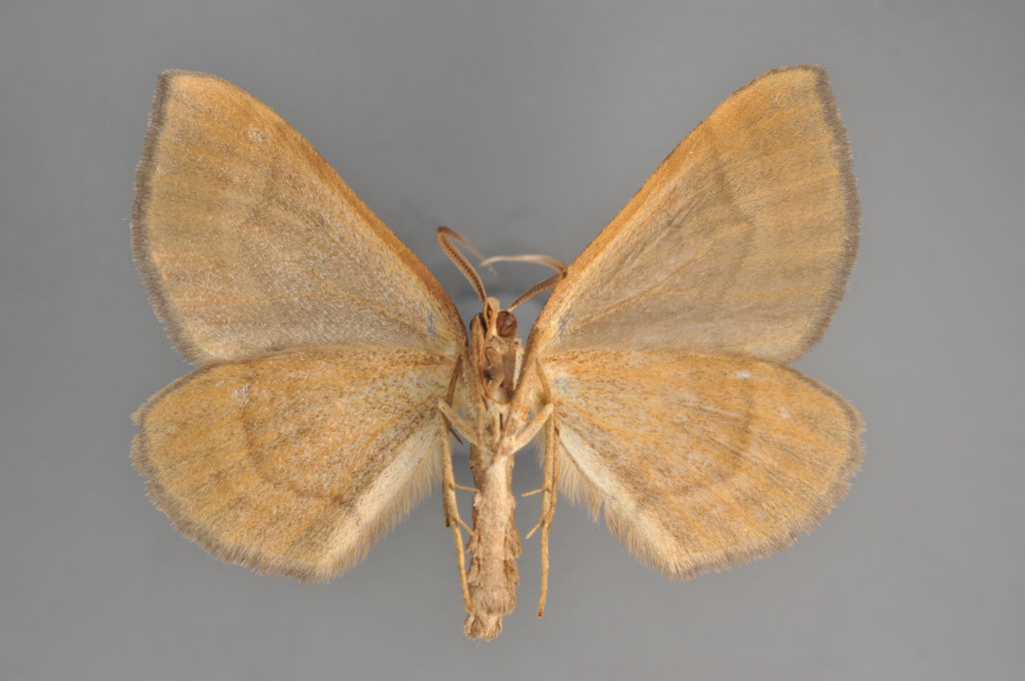
*Scotopteryx chenopodiata*, underneath

**Figure 69.**
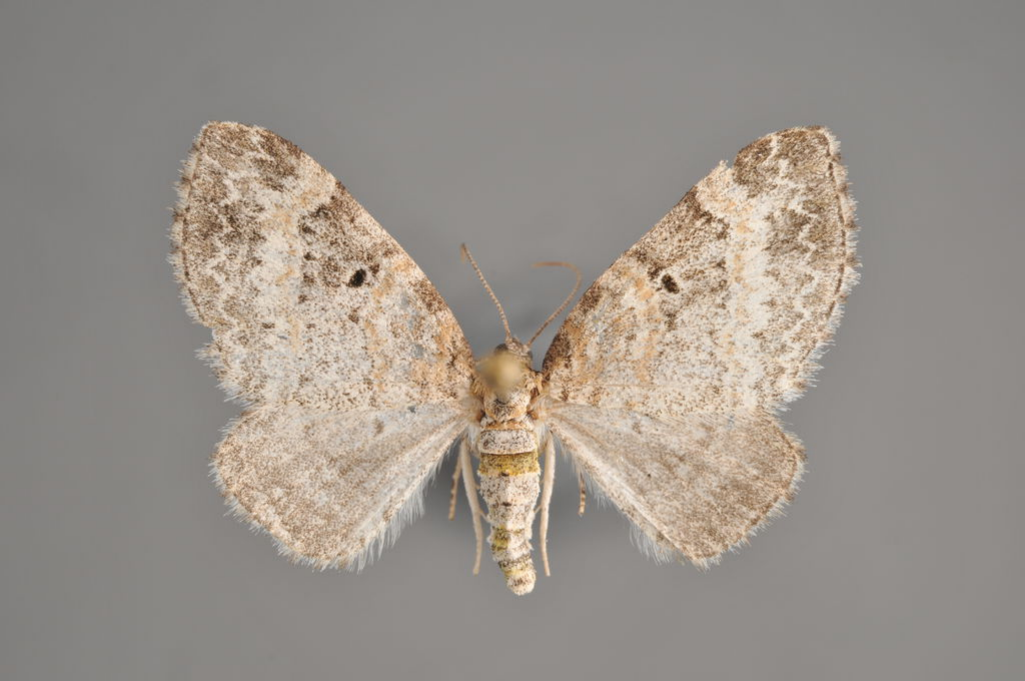
*Pterapherapteryx sexalata*, above

**Figure 70.**
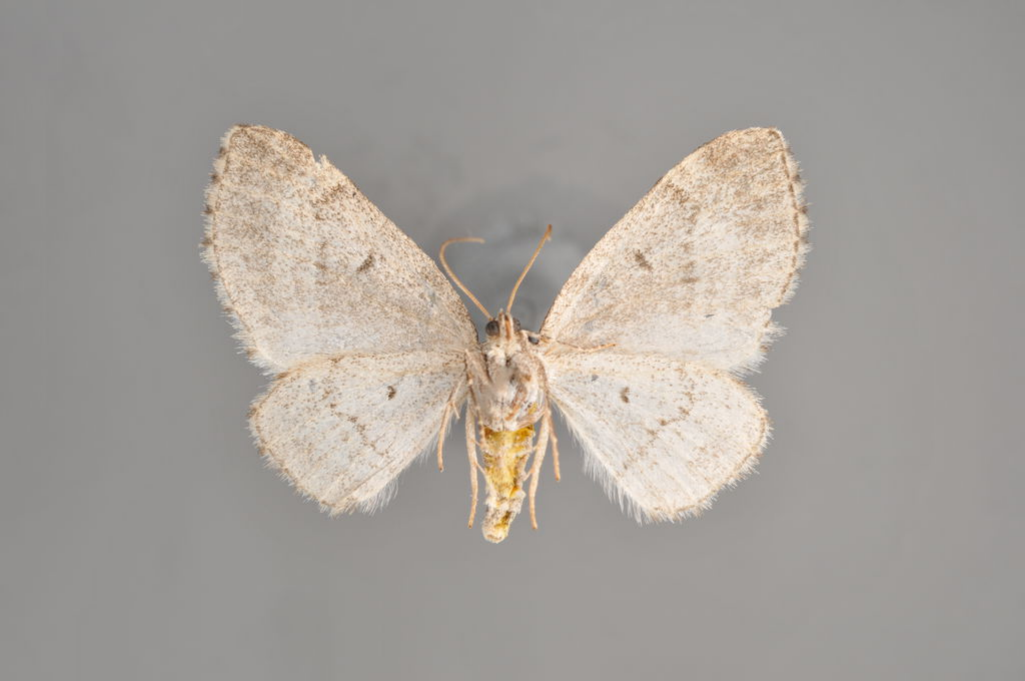
*Pterapherapteryx sexalata*, underneath

**Figure 71.**
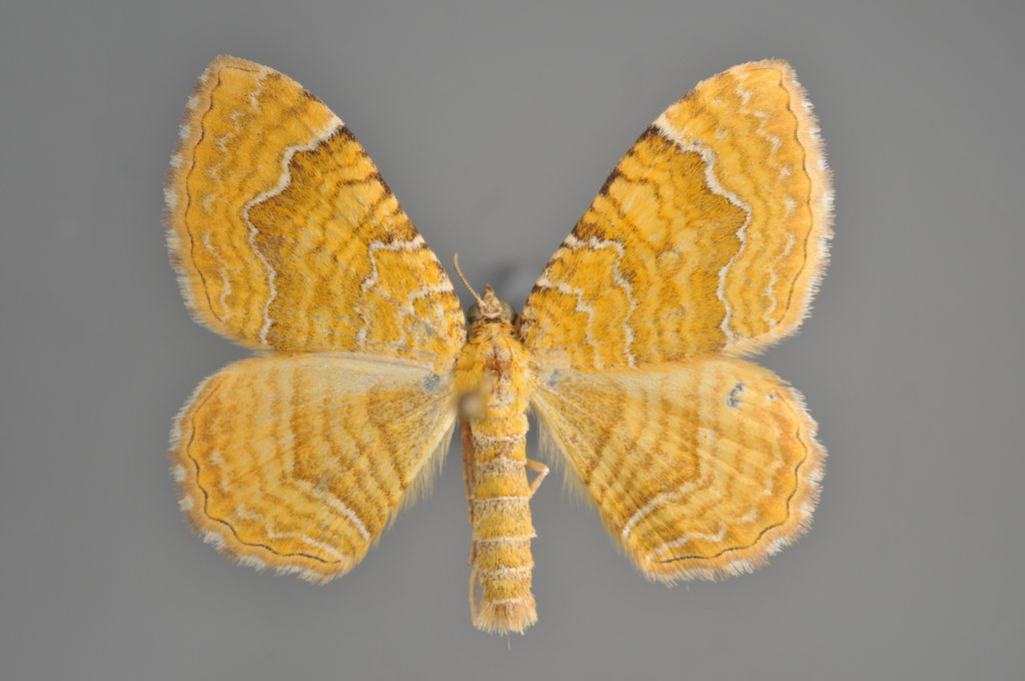
*Camptogramma bilineata*, above

**Figure 72.**
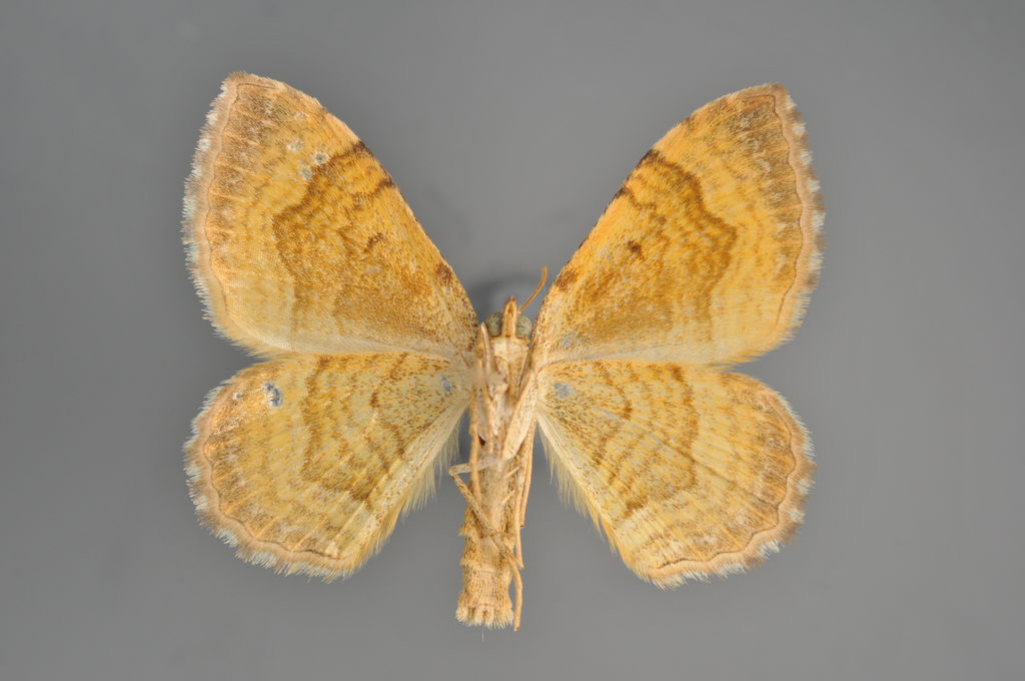
*Camptogramma bilineata*, underneath

**Figure 73.**
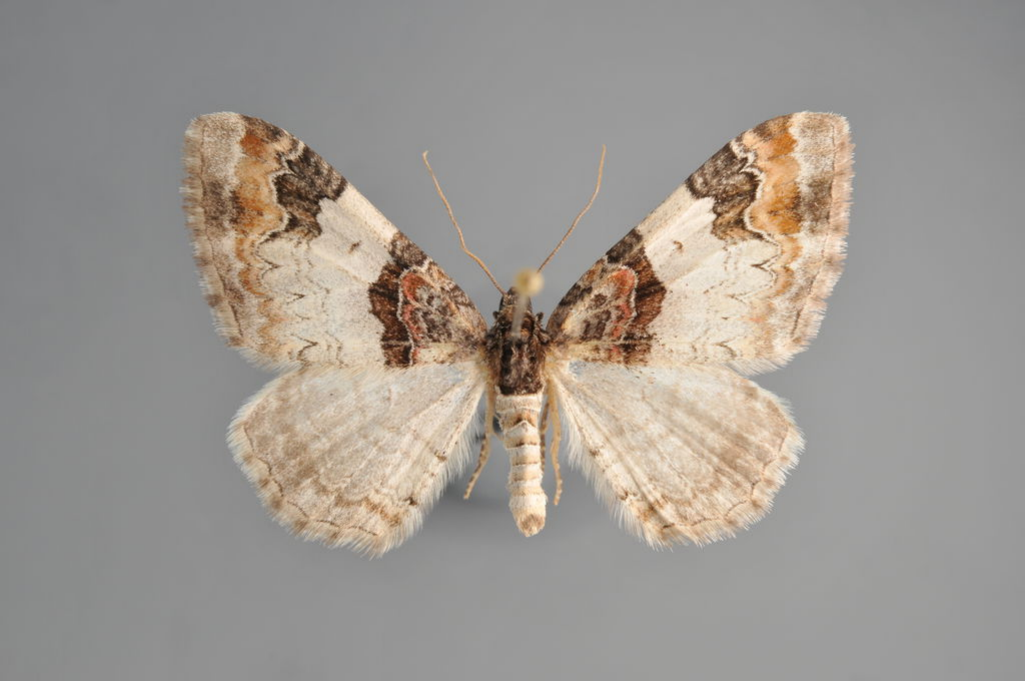
*Catarhoe cuculata*, above

**Figure 74.**
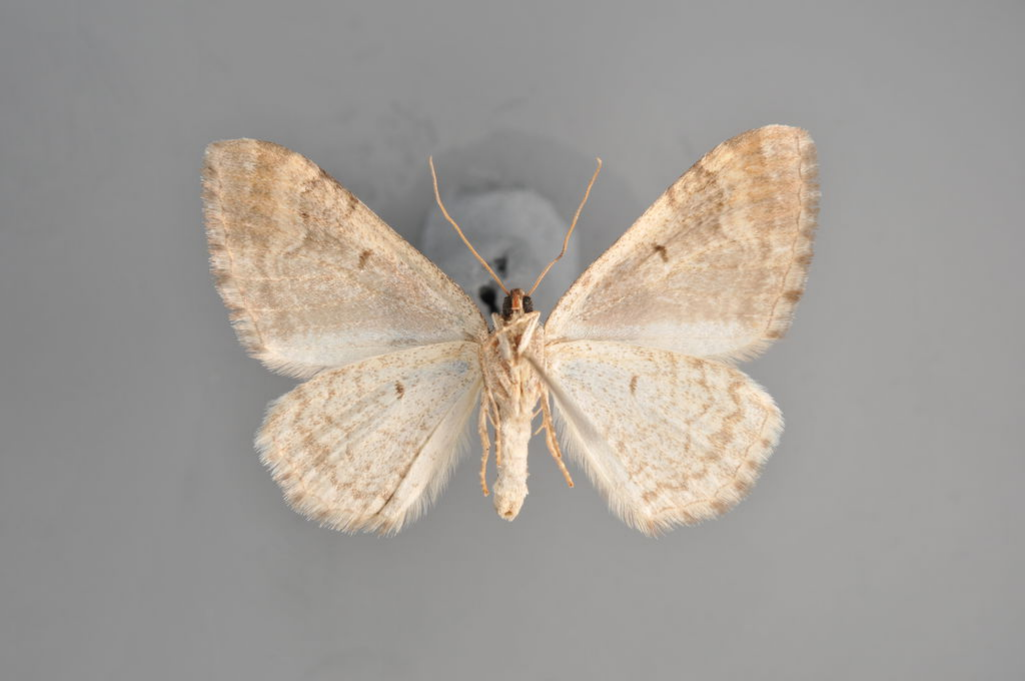
*Catarhoe cuculata*, underneath

**Figure 75.**
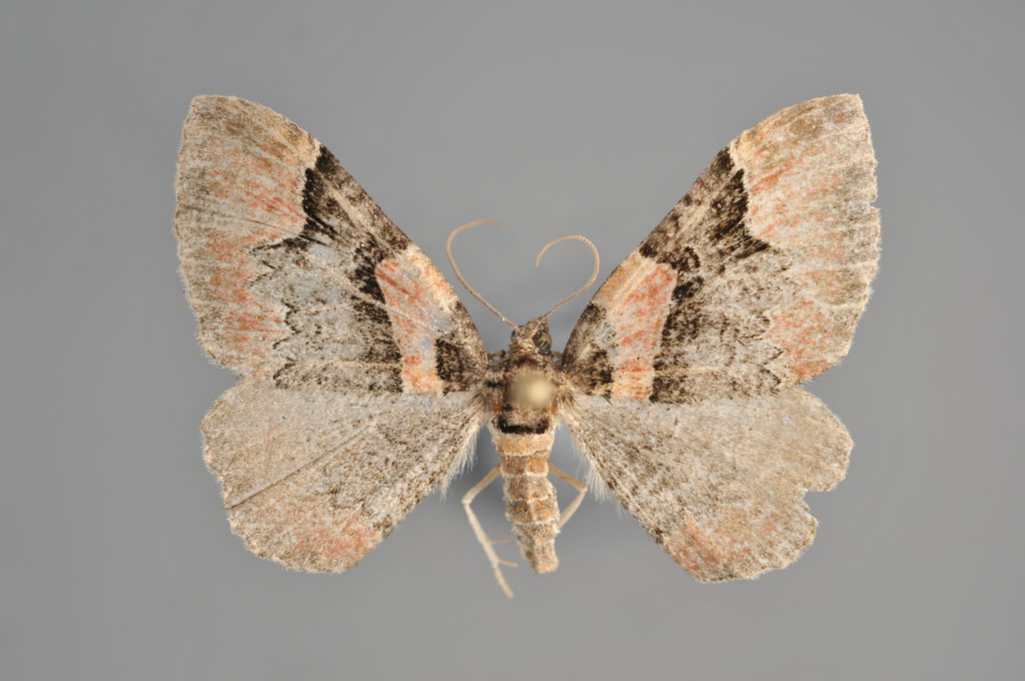
*Catarhoe rubidata*, above

**Figure 76.**
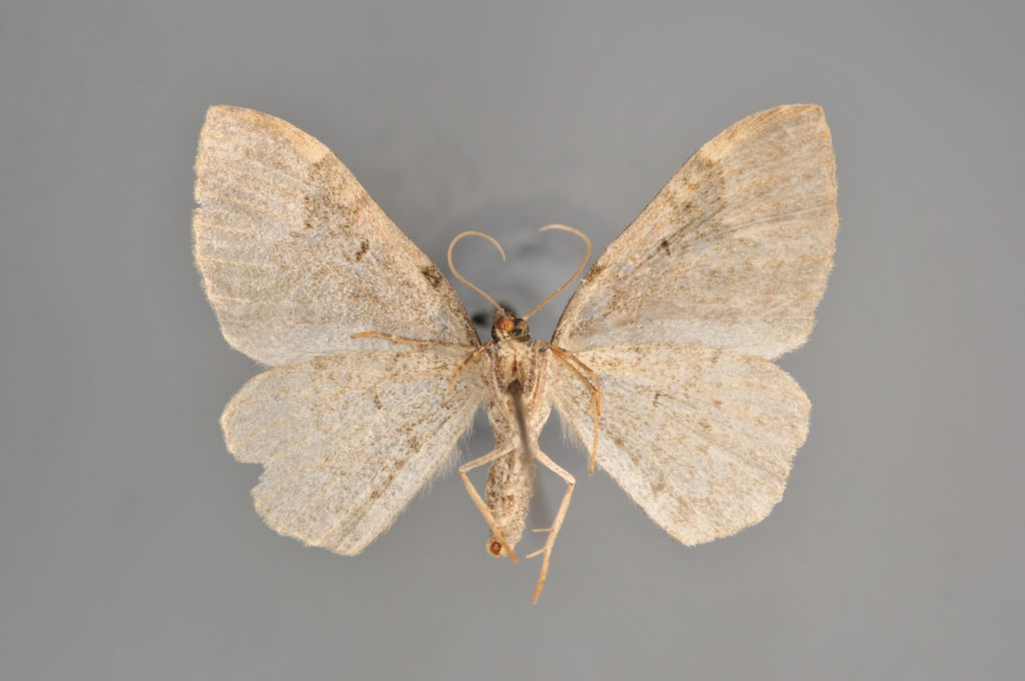
*Catarhoe rubidata*, underneath

**Figure 77.**
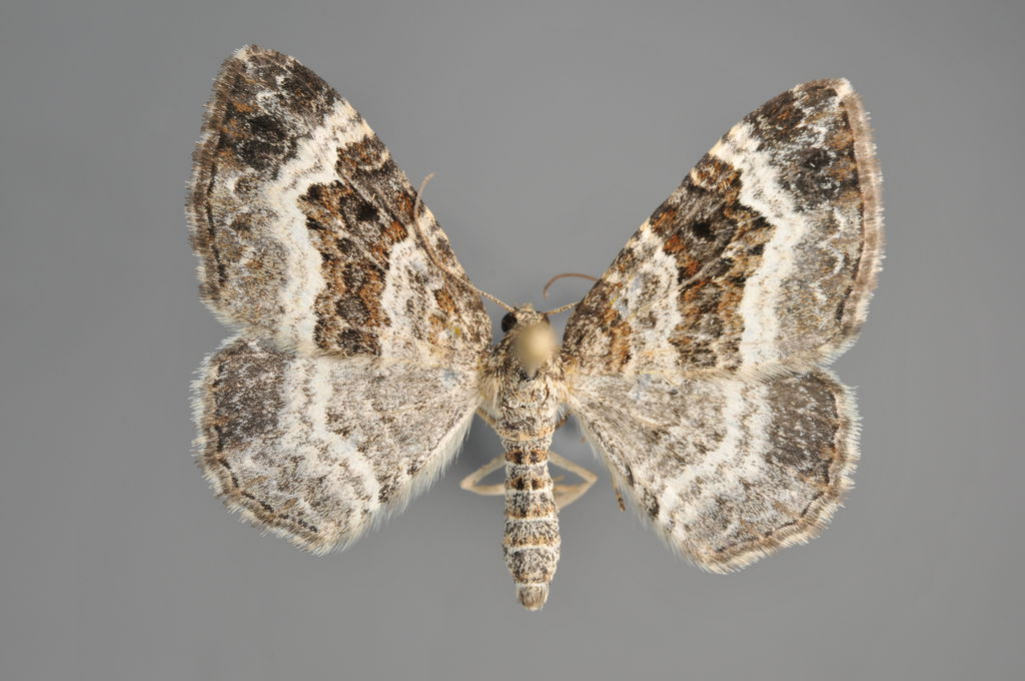
*Epirrhoe alternata*, above

**Figure 78.**
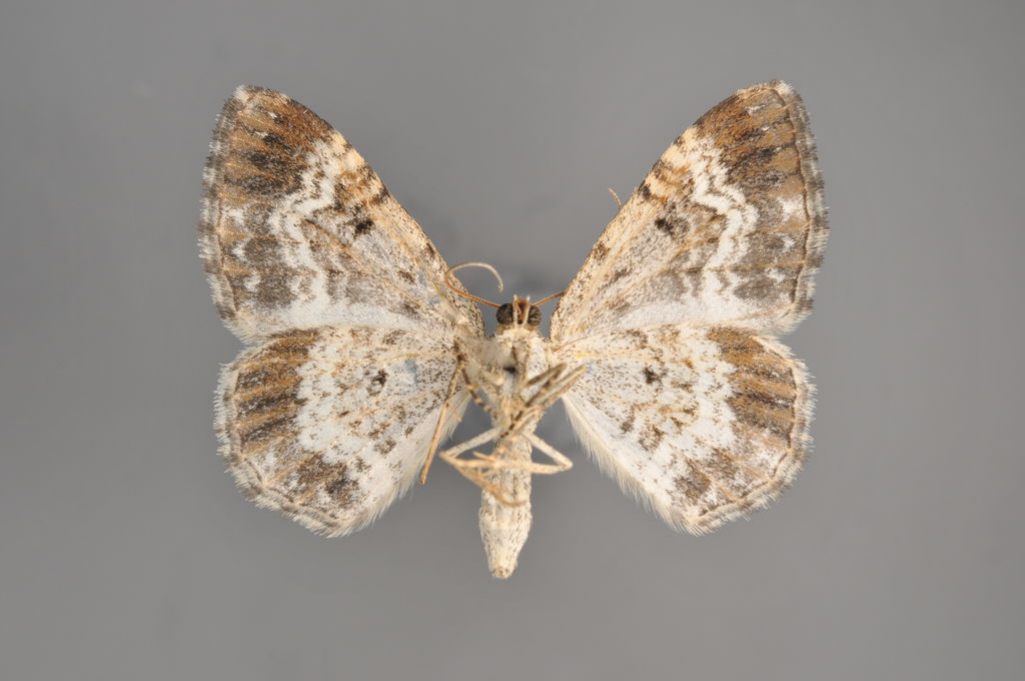
*Epirrhoe alternata*, underneath

**Figure 79.**
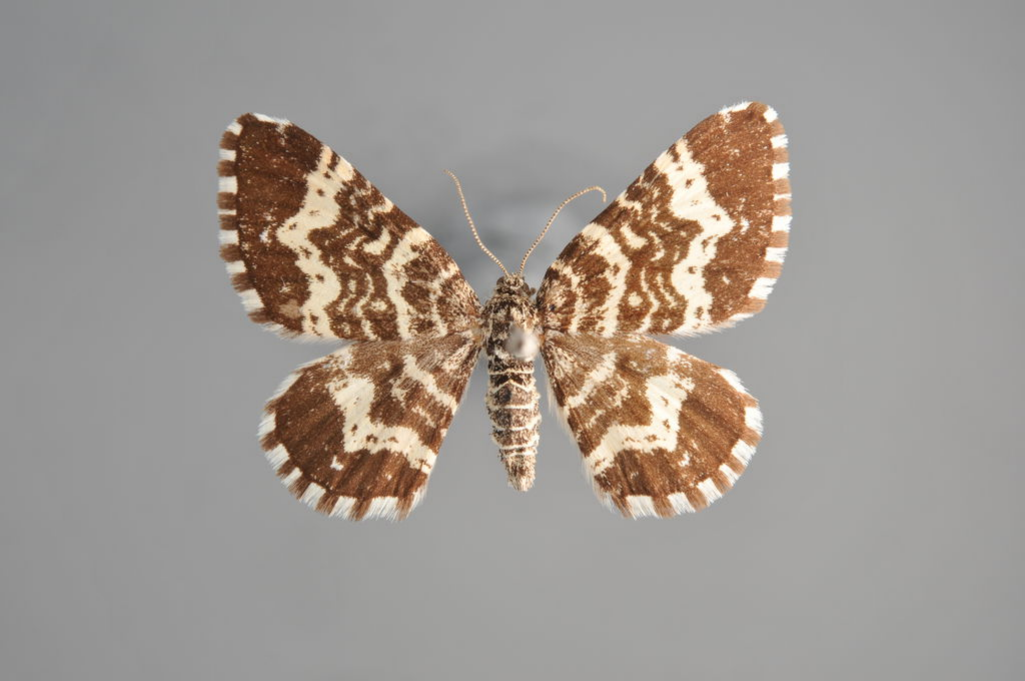
*Epirrhoe hastulata*, above

**Figure 80.**
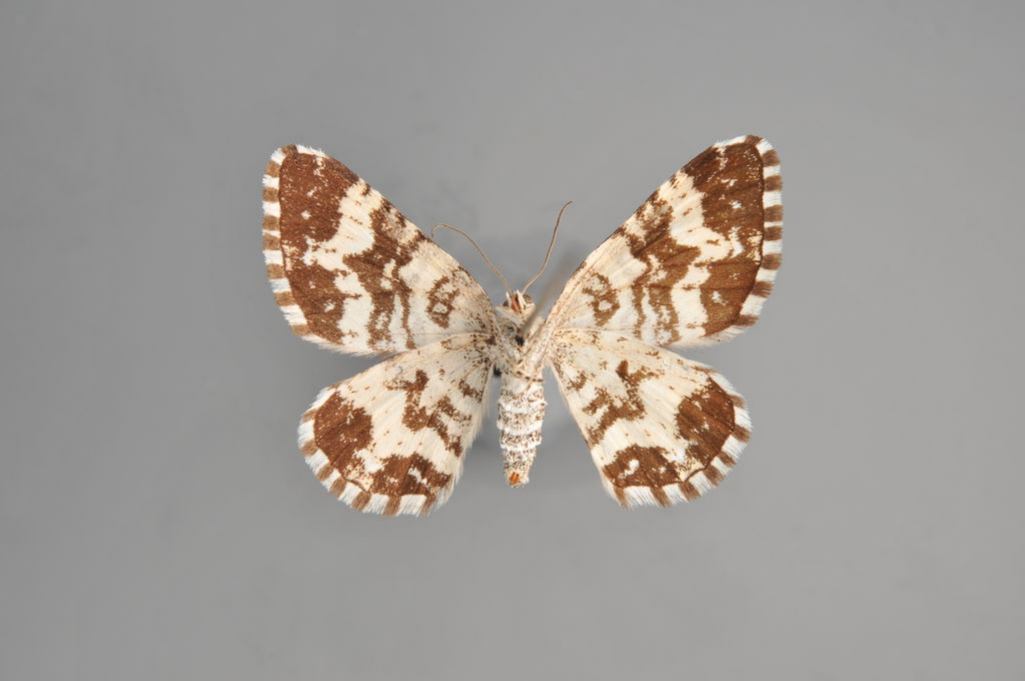
*Epirrhoe hastulata*, underneath

**Figure 81.**
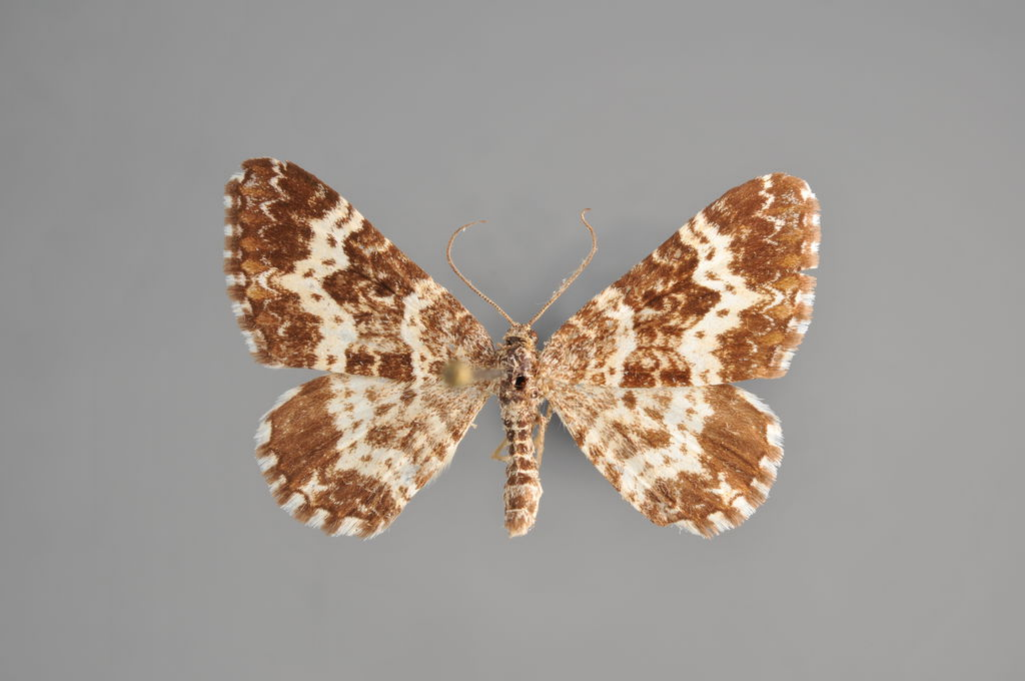
*Epirrhoe tristata*, above

**Figure 82.**
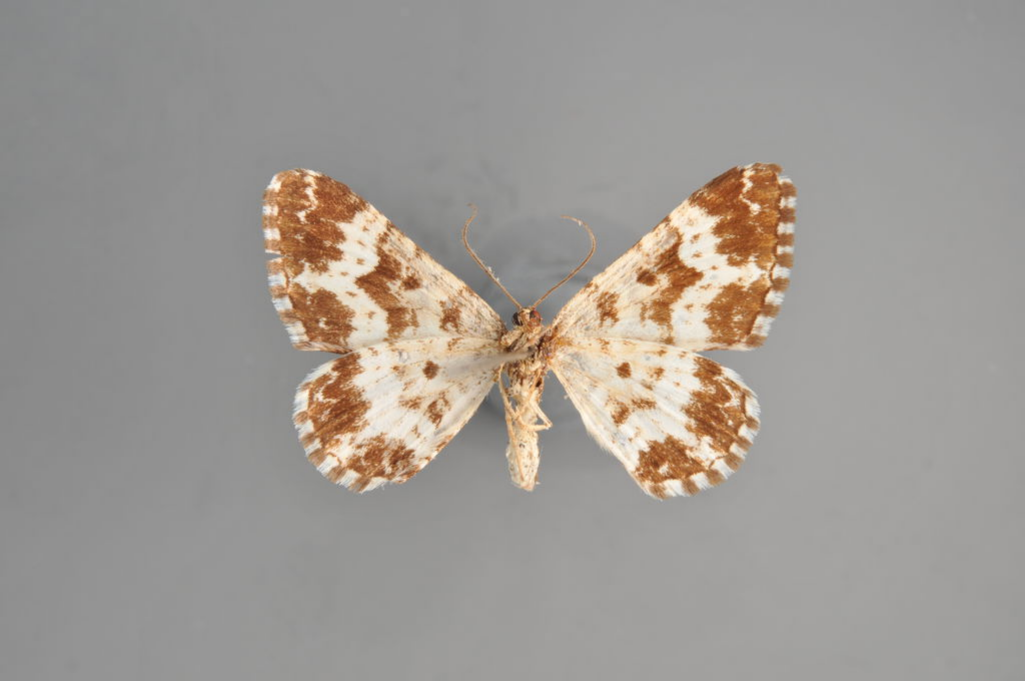
*Epirrhoe tristata*, underneath

**Figure 83.**
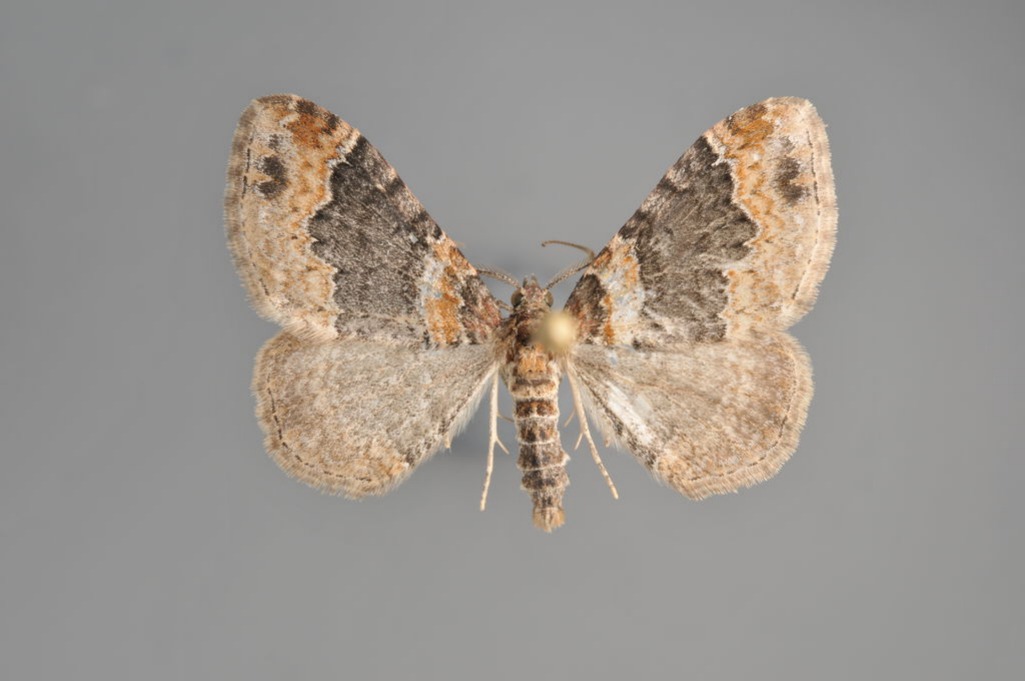
*Xanthorhoe ferrugata*, above

**Figure 84.**
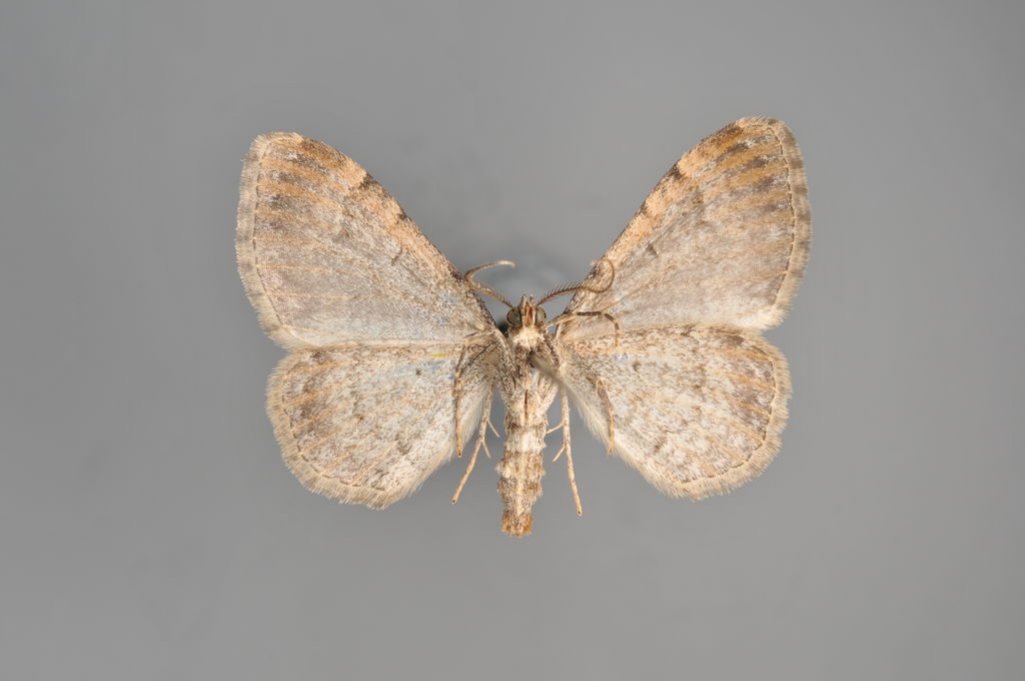
*Xanthorhoe ferrugata*, underneath
